# Financial impact of non-communicable diseases on households with older adults in india: a mixed methods study

**DOI:** 10.1186/s12877-026-07493-9

**Published:** 2026-04-18

**Authors:** Abraham Prince D E, Prakash Babu Kodali

**Affiliations:** 1 Department of Public Health and Community Medicine, Central University of Kerala, Kasaragod, Kerala India; 2 Center for Tobacco Control Research and Education, University of California, San Francisco, San Francisco, CA USA

**Keywords:** Non-communicable diseases, Catastrophic health expenditures, Impoverishment, Out-of-pocket expenditures, Healthcare, Older adults

## Abstract

**Background:**

In India healthcare expenses push > 55 million households into poverty annually. Costs for treating non-communicable diseases (NCDs) are major source of this impoverishment. We conducted this study to investigate the household economic condition, catastrophic health expenditures (CHE) and impoverishment among older adult households with NCDs.

**Methods:**

We conducted a sequential explanatory mixed-methods study with a secondary analysis of older adult households with at least one NCD (*N* = 14067), drawn from Longitudinal aging study in India (LASI), followed by qualitative interviews of 26 purposively sampled older adults living with NCDs. The proportion of CHE, impoverishment and household economic condition were estimated, and the associated factors were identified through regression analysis. Qualitative interviews were analysed thematically using framework approach.

**Results:**

Among the 14,067 households, 77.93% reported last one year healthcare use, and 16.5% (16.0%-17.0%) reported worsening economic condition. Among the households with healthcare use, 41.2%(40.4–42.0) experienced CHE, 28.4%(27.7–29.2) experienced impoverishment. NCD multimorbidity was associated with worsening household economic condition (AOR = 1.16, 95%CI = 1.05–1.27) and experiencing CHE (AOR = 1.29, 95%CI = 1.20–1.39), and those seeking both inpatient and outpatient care were more likely to be impoverished (AOR = 1.37, 95%CI = 1.23–1.52). Coverage with government health insurance did not protect households from impoverishment (AOR = 1.43, 95%CI = 1.06–1.92) compared to private health insurance. The qualitative themes revealed participant lived experiences which included (i) living with NCD as a financial nightmare, (ii) income source and financial distress, (iii) cost of care and choice of provider, and (iv) family support and lack thereof.

**Conclusions:**

The NCDs exacerbate healthcare inequities with socio-economically vulnerable older adult households at greater risk of CHE, impoverishment and worsened economic condition. Public funded health insurance programmes offered limited protection compared to private schemes.

**Supplementary Information:**

The online version contains supplementary material available at 10.1186/s12877-026-07493-9.

## Introduction

The non-communicable diseases (NCD) including cardiovascular diseases, hypertension, stroke, chronic respiratory diseases, cancer, diabetes mellitus and kidney diseases account for more than three fourth of annual deaths globally [1]. The prevalence of morbidity and mortality associated with NCD are high in low-middle income countries [2] and almost 8 out of 10 deaths attributable to NCDs occur in these countries [1]. In India two-third of annual mortality is attributed to NCD across all age categories, with a higher vulnerability among the older individuals aged 60 years and above [3]. Given that NCDs share common risk factors including ageing, NCD multimorbidity is more likely to occur among older individuals. More than 50% of older adults in India are diagnosed with at least one NCD, and NCD multimorbidity is estimated upwards of 25% [4, 5]. With the older adult population projected to be one-fifth of the Indian population by 2050, this burden of NCD morbidity is expected to further increase [6]. More than 55 million Indian households are pushed into poverty due to expenses associated with healthcare costs annually, and costs attributable to NCDs account for a substantial portion of this impoverishment [7]. Older adult households defined as the households with at least one older individual (≥ 60 years) as a primary resident, are particularly vulnerable to economic impact of NCDs and associated healthcare costs.

Population ageing coupled with high prevalence of NCDs among older individuals negate the progress in attaining the Universal Health Coverage targets across equity in access, quality healthcare delivered, and financial risk protection [8]. Owing to their chronic nature, NCDs result in high volume, high value care often resulting in catastrophic healthcare expenditures (CHE) for the households [7, 9]. Individuals treated for NCDs like diabetes, hypertension, cardiovascular disease, stroke have over 30% risk of being pushed to poverty, while living with cancer increases the risk of impoverishment by over 160% [5, 10]. Further, older adults with multimorbid conditions are less likely to receive affordable, high quality healthcare services reinforcing disparities in health outcomes [10, 11]. Studies on financial impact of NCDs reported that type of care (inpatient vs. outpatient) [12], place of residence (urban vs. rural), wealth index, place of hospitalization [13], and insurance coverage impacts the household’s risk of being pushed into catastrophic health expenditures and subsequent impoverishment [12]. Further, this risk aggravates in presence of NCD multimorbidity for households with older persons [5, 9, 14, 15]. Supplementary file 1, item S1 outlines the study’s conceptual framework.

Studies examining CHE and impoverishment attributed to NCDs are limited by their focus on households utilizing healthcare either in an inpatient or outpatient settings. However, a substantial proportion of individuals/households living with NCDs have been documented to forgo healthcare, use previous prescriptions, miss out on follow-ups and often get excluded [16, 17]. Subjective socioeconomic status has been documented to be a predictor for economic status and changes in health outcomes over time [18–20]. The household economic condition (i.e., subjective perception of betterment or worsening of the household’s economic condition over the years) along with CHE and impoverishment measures could provide a comprehensive picture of the financial impact of non-communicable diseases among older adult households. Studies exclusively focusing on financial impact of living with NCDs among older adults and their life-experiences are limited in Indian settings.

We conducted this study to investigate the household economic condition, catastrophic health expenditures and impoverishment among the older adult households with NCDs. We examined the impact of socio-economic status, insurance coverage, NCD status, and type of care received on household economic condition, CHE and impoverishment, and explored the lived experiences of older adults on financial impact of living with NCDs. By examining the household economic condition of older adult households affected by NCDs, we offer insights into the long-term financial impact of NCDs, and expand on the previous evidence that focused primarily on participants accessing inpatient or outpatient care. Furthermore, our exploration of the lived experiences of older adults with NCDs provides a firsthand account of the complex interrelationships between household finances and NCD care, and the critical roles of income sources and family support in navigating life with NCDs.

## Methodology

### Study design and setting

We conducted a mixed-methods study employing a sequential explanatory design [21, 22]. The initial quantitative phase was a secondary analysis of nationally representative sample from the Longitudinal Ageing Study in India (LASI) wave 1 (2017-18) to assess the household economic condition, catastrophic health expenditure, and impoverishment among the older adult households with NCDs. The LASI survey was conducted as a joint initiative by International Institute for Population Sciences (IIPS), Harvard T.H. Chan School of Public Health, Boston and the University of Southern California to study ageing in India. The LASI survey participants are adults aged 45 and above and their spouses, sampled through a multistage stratified area probability cluster sampling design. LASI dataset contains a representative sample of 72,250 participants from 42,949 households sampled across all Indian states. Our analysis focused specifically on households in which older adults (≥ 60 years) were living with NCDs. The detailed methodology of LASI survey is provided in the survey reports and supporting documents [6]. 

We followed up the survey analysis with an exploratory qualitative phase in which we conducted in-depth interviews in Tamil Nadu. The state of Tamil Nadu was purposively chosen due to (i) Tamil Nadu’s position as a high epidemiological transition level state with > 50% of disease burden attributed to NCDs [23], (ii) high average life-expectancy (> 72 years), and (iii) logistical feasibility of conducting qualitative research (language of the interviews, budgetary constraints, and geographical location of the study team). Further, Tamil Nadu is also at the forefront with programmes like “Makkalai Thedi Maruthuvam” which leverage grassroots healthcare infrastructure to provide NCD care to older adults with limited mobility. We conducted in-depth interviews of purposively sampled older adults living with NCDs in Tamil Nadu, to understand their lived experiences on financial impact of NCDs. Our survey analysis guided the qualitative data collection, and the triangulation of quantitative and qualitative findings was done in the interpretation phase.

### Sample size and sampling process

**Fig. 1:**
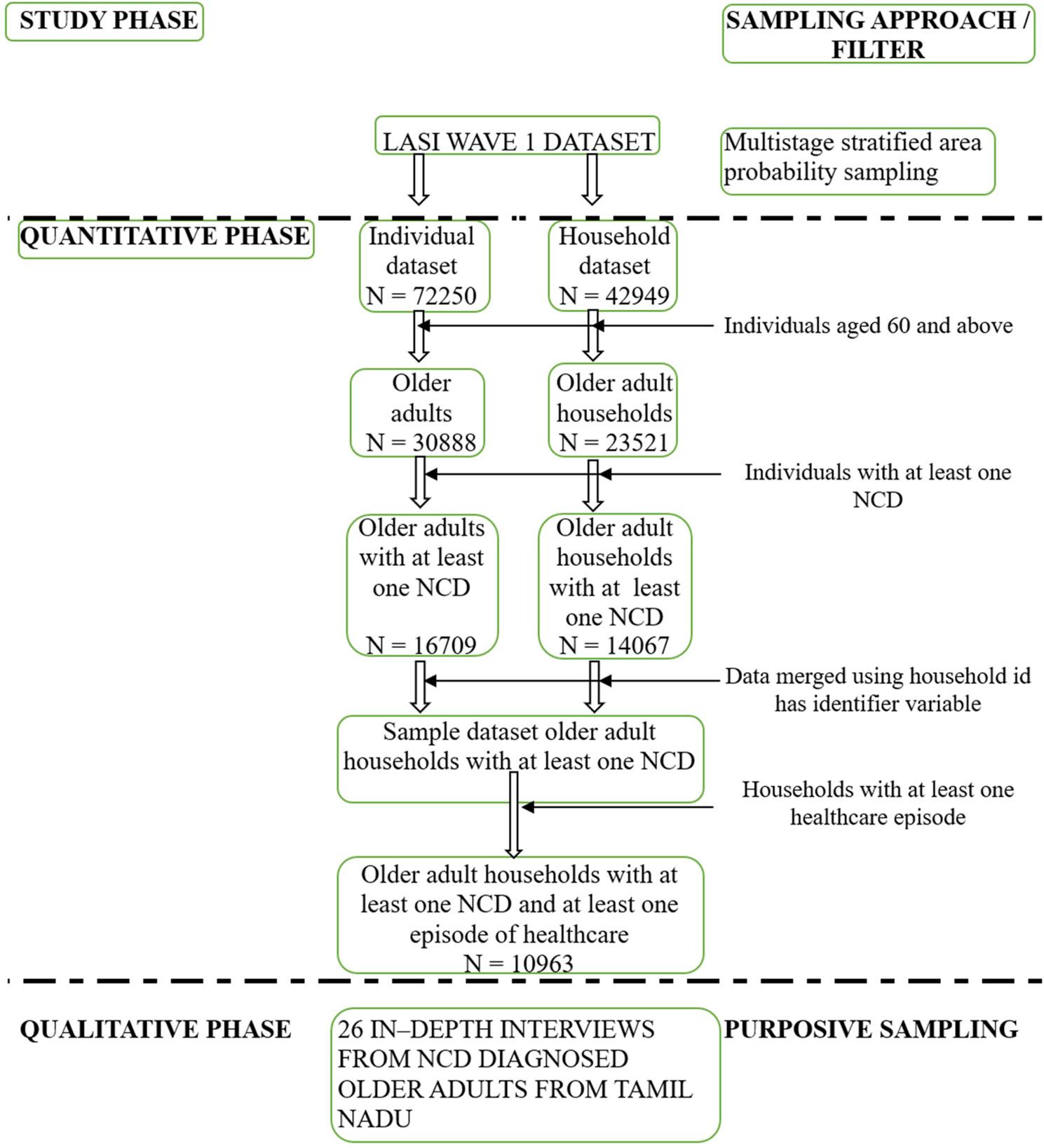
Outline of study design and sampling process. LASI= Longitudinal Ageing Study in India; NCD=Non-Communicable Diseases

The study sample for quantitative phase was a subsample derived from the LASI survey sample. The LASI surveyed a total of 42,949 households identified employing a multistage stratified area probability sampling approach [6]. Among the total households, 18,948 did not have any older persons (i.e., aged 60 years and above) and were excluded. Further, older adult households without a diagnosis of NCD (*n* = 9445) were excluded resulting in our analytic sample comprising of 14,067 households, representing 16,709 older adults living with at least one NCD (Mean age = 69.20 ± 7.35 years). Among the 14,067 households, 77.93% (*n* = 10963) reported at least one episode of any healthcare use (i.e., inpatient care or outpatient care or both) within the last one year from the survey, comprising our study sample for assessment of catastrophic health expenditure and impoverishment (Fig. 1).

In qualitative phase we purposively sampled 26 older adults living with non-communicable diseases (mean age = 67.80 ± 7.16 years) from Tamil Nadu, India. The sample size was determined by data saturation, assessed through the point at which the interviews reached information redundancy [24]. To ensure maximum variation, the participants were selected across sex, socio-economic status, insurance coverage, and non-communicable disease conditions.

### Study variables

The financial impact of living with non-communicable diseases was assessed based on three outcomes (i) household economic condition, (ii) catastrophic health expenditure, and (iii) impoverishment.

Household economic condition was computed as a single self-reported item on economic status of the household “*Would you say your household’s overall economic condition has improved*,* stayed about the same*,* or worsened*,* compared to two years ago?*” with the responses “improved”, “stayed same”, or “worsened”. The household’s economic condition was considered “worsened” if the participant reported that his/her household’s overall economic condition worsened in the past two years.

The catastrophic health expenditures and impoverishment were computed among the households reporting at least one episode of healthcare use within one year preceding the survey. First the out-of-pocket health expenditures for the outpatient (OP) care, inpatient (IP) care and total health expenditures were computed. Further, other household expenditures including the total food expenditure, total non-food expenditure, and expenditures on consumables and durables were computed. These measures were further used to calculate the CHE & Impoverishment using the Capacity to Pay (CTP) approach [25]. The CTP approach employs validated methods to compute household specific subsistence expenditure, and capacity to pay based on the household size, food and non-food expenditures. We outlined the process of computing CTP, CHE and impoverishment levels in supplementary file 1, item S2.

We used the 40% cutoff to categorize CHE, wherein the households reporting health related expenses of more than 40% of CTP were considered to be incurring CHE [25]. Further, we also assessed CHE at the 25% and 30% thresholds, and presented the findings in supplementary file 2 to indicate towards the robustness of using 40% cutoff to categorize CHE. The impoverishment was defined as household expenditure equal to/higher than the subsistence spending but lesser than the subsistence spending after deducting the healthcare expenditure [25]. Poor households incurring any health expenditure were also considered impoverished [26]. 

Independent variables studied include household socio-demographic characteristics such as (i) sex of the household head (male/female), (ii) caste of the household head (other backward castes, scheduled castes, scheduled tribes, and others), (iii) income source (i.e., agriculture & non-agricultural business, individual salary, and government subsidies and other income), and (iv) place of residence (urban/rural). Economic status of the household was based on the variable wealth index which was recoded to responses (i) poor, (ii) middle and (iii) rich. Non-communicable disease status of the household was defined as presence of at least one older person with NCD, with the NCD status captured as a binary variable (i.e., single NCD or NCD multimorbidity). The type of care utilized in the last one year was defined as (i) inpatient care, (ii) outpatient care, (iii) both inpatient and outpatient care, and (iv) no care. The state of residence was categorized based on the epidemiological transition level (ETL) of the state with the categories (i) Low ETL [Bihar, Jarkhand, Uttar Pradesh, Rajasthan, Meghalaya, Assam, Chhattisgarh, Madhya Pradesh, Odisha], (ii) Lower Middle ETL [Arunachal Pradesh, Mizoram, Nagaland, Uttarakahnd, Gujarat, Tripura, Manipur], (iii) Higher Middle ETL [Haryana, Delhi, Telangana, Andhra Pradesh, Jammu and Kashmir, Karnataka, West Bengal, Maharashtra, and Union territories other than Delhi], and High ETL [Himachal Pradesh, Punjab, Tamil Nadu, Goa, and Kerala], reflecting the chronic disease distribution across the states [23]. We provided the details of computing independent variables in supplementary file 1, item S3.

### Data collection

The LASI survey was conducted through structured interview employing validated survey questionnaire by the trained field investigators. The specific details of data collection processes, data management and quality control are provided in LASI survey report [6]. We obtained the deidentified unit-level survey data from the International Institute of Population Sciences (IIPS), the nodal agency for implementing LASI through a formal request.

In the qualitative phase, semi-structured in-depth interview guide was employed to conduct participant interviews. The interview guide was developed based on the review of literature, and theoretical models on wellbeing and healthcare utilization [9, 16, 27]. The interview guide’s content was validated through expert judgement by subject experts who were not part of the study. The face validity was established by piloting the interview guide with three participants. The interview guide was translated to Tamil and back translated to check for consistency. The first author, who is a native Tamil speaker conducted the interview. The interviews were conducted at participant’s home and were audio recorded with a prior consent from the participants.

### Data preparation and analysis

#### Quantitative data analysis

Prior to analysis the data preparation phase involved extraction of the study sample from the larger LASI data set, generating code books, missing data analysis, generating study variables and merging household and individual data files. Recalibrated sample weights were applied to account for the complex sample design. Our study was among a subsample of older adult households with NCDs derived from the larger LASI sample. Accordingly, we recalibrated the sample weights by multiplying the original sample weights by the inverse probability of selection for older adult households with NCDs [28]. The outcomes household economic condition, CHE and impoverishment due to healthcare expenses were descriptively analyzed to estimate weighted proportions. Multinomial logistic regression was used to assess the factors associated with household economic condition. Binary logistic regression models were developed to assess the factors associated with (i) catastrophic health expenditure, and (ii) impoverishment. Socio-economic variables, NCD status, healthcare seeking of household member, and ETL level of state of residence were the independent variables across logistic regression models. Adjusted odds ratios (AOR) were computed to adjust for the confounding effect, and variance inflation factor was computed to check for presence multicollinearity.

#### Qualitative data analysis

The semi-structured interviews were transcribed verbatim and translated from Tamil to English. The interviews were analyzed using thematic analysis employing the framework approach advocated by Braun and Clarke [29]. The interviews were first familiarized and an initial free list of codes were developed. The interviews were further read and coded both deductively (based on conceptual framework) and inductively to develop semantic themes. To ensure consistency in findings, authors independently read the transcripts and coded them. The codes were listed and condensed using Microsoft Excel^®^.

## Results

Among the survey sample, close to half of the households (47.7%) reported having an older adult living with self-reported NCD multi-morbidity. Around four in five (*n* = 10963, 79.4%) reported availing some form of health care in the last one year, and 74.2% were not at all covered by any health insurance (Table 1).

**Table 1:** Characteristics of older adult households studied (*n* = 14067)

VARIABLES	PERCENTAGE
Sex of household head	
Male	80.5
Female	19.5
Caste of household head	
Other backward class	45.8
Scheduled caste	18.5
Scheduled tribe	5.7
Others^a^	30.0
Education of household head	
No schooling	33.5
Up to middle school	37.0
Up to diploma/higher secondary	20.0
Graduate and above	9.0
Income source	
Agriculture & Non-agricultural business	38.3
Individual salary	48.6
Government subsidies and other income	13.1
Wealth index	
Poor	38.9
Middle	21.3
Rich	39.9
Residence	
Urban	35.3
Rural	64.7
Epidemiological transition level	
Low ETL	34.6
Lower middle ETL	6.2
Higher middle ETL	44.0
High ETL	15.1
Health Insurance coverage	
Government	22.2
Private	3.7
Not covered	74.2
NCD status	
Single NCD	52.3
NCD multimorbidity	47.7
Type of care	
OP care only	56.3
IP care only	3.9
Both OP & IP care	19.3
Care not availed	20.6

ETL: Epidemiological Transition Level; Low ETL- Bihar, Jarkhand, Uttar Pradesh, Rajasthan, Meghalaya, Assam, Chhattisgarh, Madhya Pradesh, Odisha; Lower Middle ETL- Arunachal Pradesh, Mizoram, Nagaland, Uttarakahnd, Gujarat, Tripura, Manipur; Higher Middle ETL- Haryana, Delhi, Telangana, Andhra Pradesh, Jammu and Kashmir, Karnataka, West Bengal, Maharashtra, and Union territories other than Delhi; High ETL- Himachal Pradesh, Punjab, Tamil Nadu, Goa, and Kerala. NCD: Non-Communicable Diseases. OP: Outpatient care, IP: Inpatient care; ^a^Others included the caste groups which were not reported to be scheduled castes, scheduled tribes or other backward castes. The self-reported diagnosis with hypertension, diabetes, cancer, chronic lung diseases, chronic heart diseases, stroke, arthritis, neurological problems, high cholesterol or other chronic conditions were included in assessment of NCD status. Note: The percentages reported are weighted percentages

### Household economic condition, catastrophic health expenditure and impoverishment

**Fig. 2:**
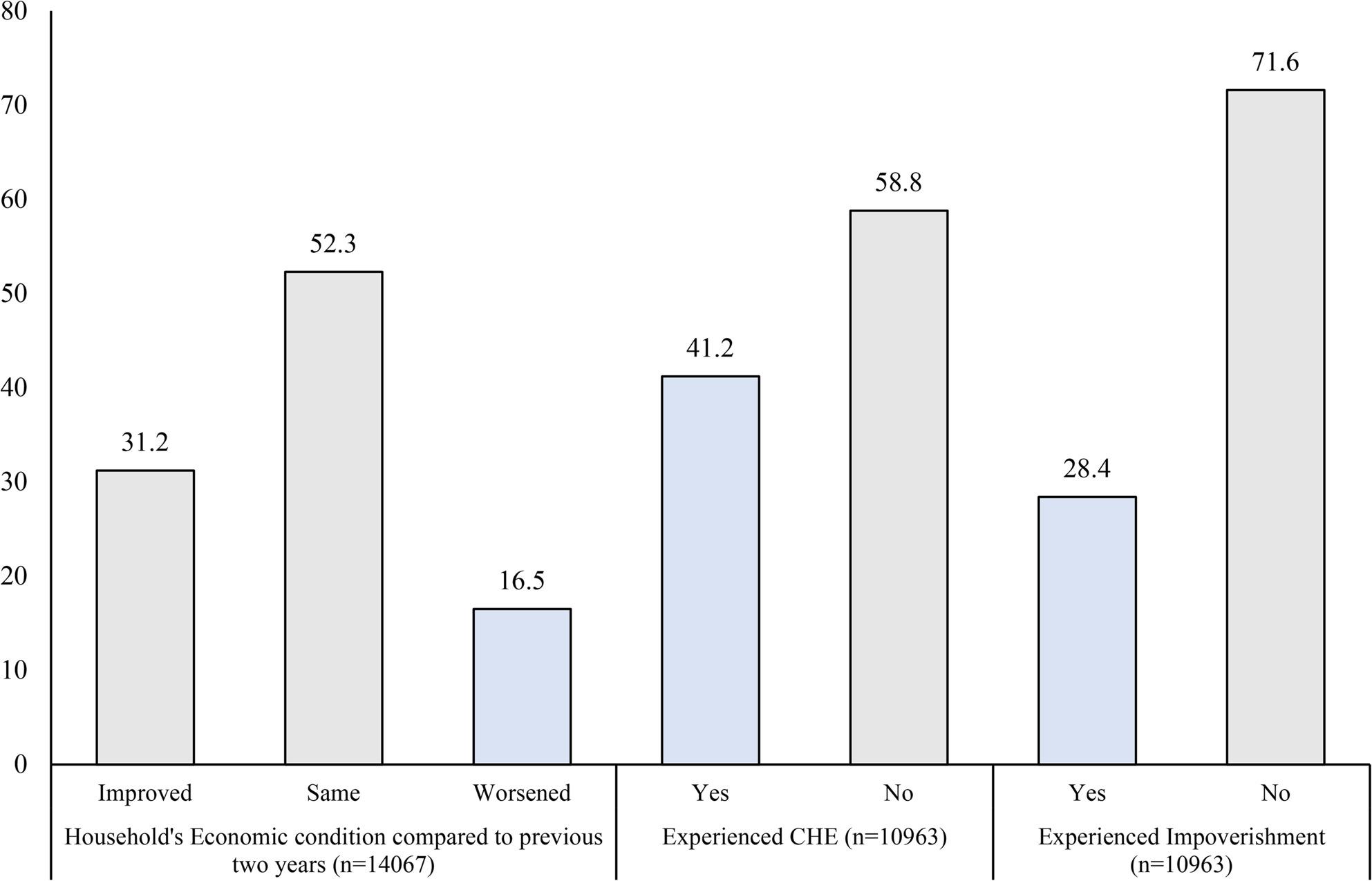
Household economic condition, catastrophic health expenditure and impoverishment among older adult households with NCD. Footnotes: CHE – Households having healthcare expenditure more than 40% of their capacity to pay. Impoverishment – non-poor households where the non-health expenditure is lesser than the subsistence expenditure and poor households incurring health expenditures. Household economic condition – self reported by the respondent

Half of the households (52.3%) reported that the household economic condition remained same compared to previous years. Around 16.5% (16.0% – 17.0%) of all older adult households living with NCD reported worsening of household’s economic condition. Among the households who availed some form of care, 41.2% (40.4% – 42.0%) experienced catastrophic health expenditures, and 28.4% (27.7% – 29.2%) experienced impoverishment (Fig. 2).

### Factors associated with household economic condition

The multinomial logistic regression yielded that, among the older adult households studied, female headed households (AOR = 1.76, 95% CI = 1.57–1.97), scheduled caste households (AOR = 1.67, 95%CI = 1.48–1.90) and those reporting government subsidies and benefits as an income source (AOR = 2.51, 95%CI = 2.18–2.89) reported worsening of the household economic status. NCD multimorbidity (AOR = 1.16, 95%CI = 1.05–1.27) and accessing both IP & OP care (AOR = 1.90, 95%CI = 1.64–2.21) significantly increased the likelihood of worsened economic status. The households covered by government health insurance (AOR = 1.44, 95%CI = 1.20–1.73) and those ‘not covered’ by any health insurance coverage (AOR = 1.23, 95%CI = 1.03–1.46) were more likely to report the ‘household economic condition to have remained same’ compared to the households covered by private health insurance. The households from higher middle ETL group (AOR = 0.50, 95%CI = 0.45–0.56) were less likely to have the economic condition to remain same compared to households from high ETL group (Table 2).

**Table 2:** Factors associated household economic condition (*n* = 14067)

Predictor variables	HH economic condition remained same		HH economic condition Worsened	
	%	AOR (95% CI)	%	AOR(95% CI)
Gender of household head				
Female	54.0	1.35 (1.24–1.48)**	20.4	1.76 (1.57–1.97)**
Male (Ref)	52.1		15.3	
Caste				
Scheduled caste	54.2	1.32 (1.20–1.46)**	21.3	1.67 (1.48–1.90)**
Scheduled tribe	60.9	1.37 (1.18–1.60)**	11.8	0.79 (0.63–0.99)*
Others	50.7	1.16 (1.07–1.25)**	15.3	1.21 (1.08–1.35)*
Other backward class (Ref)	51.9		15.6	
Income source				
Agricultural & non-agricultural business	54.5	1.21 (1.12–1.30)**	16.9	1.34 (1.21–1.48)**
Government subsidies & benefits	52.7	1.49 (1.33–1.67)**	24.2	2.51 (2.18–2.89)**
Individual salary (Ref)	50.9		13.8	
Wealth index				
Middle	53.1	1.24 (1.13–1.35)**	14.6	1.14 (1.00-1.29)
Poor	55.9	1.70 (1.57–1.84)**	18.7	2.07 (1.86–2.31)**
Rich (Ref)	48.8		14.9	
Residence				
Rural	54.7	1.63 (1.51–1.76)**	18.8	2.35 (2.11–2.62)**
Urban (Ref)	48.4		11.8	
Epidemiological transition level(ETL)				
Low ETL	52.6	0.52 (0.46–0.58)**	16.7	0.56 (0.48–0.65)**
Lower middle ETL	51.7	0.61 (0.51–0.72)**	17.2	0.82 (0.66 − 0.1.03)
Higher middle ETL	49.6	0.50 (0.45–0.56)**	15.5	0.55 (0.48–0.63)**
High ETL	60.7		17.5	
Health insurance				
Government	55.5	1.44 (1.20–1.73)**	17.4	1.15 (0.89–1.49)
Not covered	52.0	1.23 (1.03–1.46)*	16.1	0.95 (0.75–1.22)
Private (Ref)	44.2		15.0	
NCD status				
NCD multimorbidity	50.7	0.98 (0.92–1.05)	16.8	1.16 (1.05–1.27)*
Single NCD (Ref)	54.1		16.0	
Type of care				
IP care	53.4	1.14 (0.95–1.37)	12.8	1.04 (0.79–1.36)
IP & OP care	48.0	1.13 (1.01–1.26)*	19.9	1.90 (1.64–2.21)**
OP care	53.6	1.22 (1.12–1.34)**	16.0	1.39 (1.23–1.58)**
No care (Ref)	53.5		14.5	

A single multivariate model was developed using multinominal logistic regression analysis with “Household economic condition” as the dependent variable

Dependent variable: Household Economic Condition in the previous two years: (i) Improved [ref], (ii) remained same, (ii) worsened

ETL: Epidemiological Transition Level; Low ETL- Bihar, Jarkhand, Uttar Pradesh, Rajasthan, Meghalaya, Assam, Chhattisgarh, Madhya Pradesh, Odisha; Lower Middle ETL- Arunachal Pradesh, Mizoram, Nagaland, Uttarakahnd, Gujarat, Tripura, Manipur; Higher Middle ETL- Haryana, Delhi, Telangana, Andhra Pradesh, Jammu and Kashmir, Karnataka, West Bengal, Maharashtra, and Union territories other than Delhi; High ETL- Himachal Pradesh, Punjab, Tamil Nadu, Goa, and Kerala; HH=Household, IP= Inpatient, OP=Out Patient NCD: Non-Communicable Diseases, Ref= reference category

The self-reported diagnosis with hypertension, diabetes, cancer, chronic lung diseases, chronic heart diseases, stroke, arthritis, neurological problems, high cholesterol or other chronic conditions were included in assessment of NCD status. * - *p*-value <0.05, **- *p*-value <0.001.The percentages reported are weighted percentages

### Factors associated with catastrophic health expenditure and impoverishment

The Binary logistic regression analysis indicate that CHE and Impoverishment associated with hospitalization among the households having older persons living with NCDs share similar vulnerabilities. Households headed by females experienced higher CHE (AOR = 1.22, 95% CI = 1.12–1.34) and impoverishment (AOR = 2.03, 95% CI = 1.81–2.27) compared to men headed households. Similarly, scheduled caste households had higher odds of impoverishment (AOR = 1.17, 95% CI = 1.04–1.31). Coverage by a government funded health insurance was not protective against impoverishment (AOR = 1.43, 95% CI = 1.06–1.92) compared to private health insurance coverage, while NCD multimorbidity significantly increased the odds of CHE (AOR = 1.29, 95% CI = 1.20–1.39) (Table 3).

**Table 3:** Factors associated with CHE and Impoverishment (*n* = 10963)

Variables	CHE-40		Impoverishment	
	%	AOR (95% CI)	%	AOR (95% CI)
Gender of household head				
Female	43.7	1.22 (1.12–1.34)**	34.2	2.03 (1.81–2.27)**
Male (Ref)	40.4		26.6	
Caste				
Scheduled caste	45.1	1.09 (0.99–1.20)	38.1	1.17 (1.04–1.31)*
Scheduled tribe	38.9	0.72 (0.62–0.85)**	35.5	1.10 (0.91–1.33)
Others	38.0	0.94 (0.87–1.02)	19.8	0.83 (0.74–0.93)*
Other backward class (Ref)	41.7		28.5	
Income source				
Agricultural & non-agricultural business	44.6	1.25 (1.16–1.35)**	31.1	1.54 (1.39–1.70)**
Government subsidies & others	51.2	1.81 (1.62–2.03)**	43.7	3.26 (2.84–3.75)**
Individual salary (Ref)	35.6		21.6	
Wealth index				
Middle	41.0	0.99 (0.90–1.09)	22.0	3.52 (3.08–4.02)**
Poor	40.3	1.14 (1.06–1.24)**	56.4	22.78(20.19–25.70)**
Rich (Ref)	41.4		7.6	
Residence				
Rural	46.5	2.01 (1.85–2.18)**	34.9	4.38 (3.91–4.90)**
Urban (Ref)	30.1		14.1	
Epidemiological transition level				
Low ETL	43.6	1.37 (1.22–1.53)**	34.5	1.20 (1.04–1.40)*
Lower middle ETL	23.5	0.62 (0.51–0.75)**	14.3	0.66 (0.51–0.86)*
Higher middle ETL	42.8	1.37 (1.22–1.54)**	25.8	1.23 (1.06–1.43)*
High ETL (Ref)	35.7		22.3	
Health insurance				
Government	38.9	1.19 (0.98–1.46)	27.8	1.43 (1.06–1.92)*
Not covered	42.2	1.33 (1.10–1.60)**	28.7	1.39 (1.05–1.85)*
Private(Ref)	31.0		14.3	
NCD status				
NCD multimorbidity	43.1	1.29 (1.20–1.39)**	23.7	0.97 (0.88–1.06)
Single NCD (Ref)	39.0		32.1	
Type of care				
IP care	40.9	1.52 (1.29–1.78)**	27.7	1.11 (0.89–1.38)
IP & OP care	61.3	3.31 (3.05–3.59)**	25.0	1.37 (1.23–1.52)**
OP care (Ref)	34.1		29.0	

The table reports the findings from two separate binary logistic regression models with the binary outcome variables (i) Catastrophic health expenditure, and (ii) impoverishment respectively

Dependent Variables: Catastrophic Health Expenditure (CHE)= No (Ref), Yes; Impoverishment=No (Ref), Yes

ETL: Epidemiological Transition Level; Low ETL- Bihar, Jarkhand, Uttar Pradesh, Rajasthan, Meghalaya, Assam, Chhattisgarh, Madhya Pradesh, Odisha; Lower Middle ETL- Arunachal Pradesh, Mizoram, Nagaland, Uttarakahnd, Gujarat, Tripura, Manipur; Higher Middle ETL- Haryana, Delhi, Telangana, Andhra Pradesh, Jammu and Kashmir, Karnataka, West Bengal, Maharashtra, and Union territories other than Delhi; High ETL- Himachal Pradesh, Punjab, Tamil Nadu, Goa, and Kerala; HH=Household, IP= Inpatient, OP=Out Patient NCD: Non-Communicable Diseases, Ref= reference category

The self-reported diagnosis with hypertension, diabetes, cancer, chronic lung diseases, chronic heart diseases, stroke, arthritis, neurological problems, high cholesterol or other chronic conditions were included in assessment of NCD status. * - *p*-value <0.05, **- *p*-value <0.001. Note: The percentages reported are weighted percentages

### Qualitative results

**Fig. 3:**
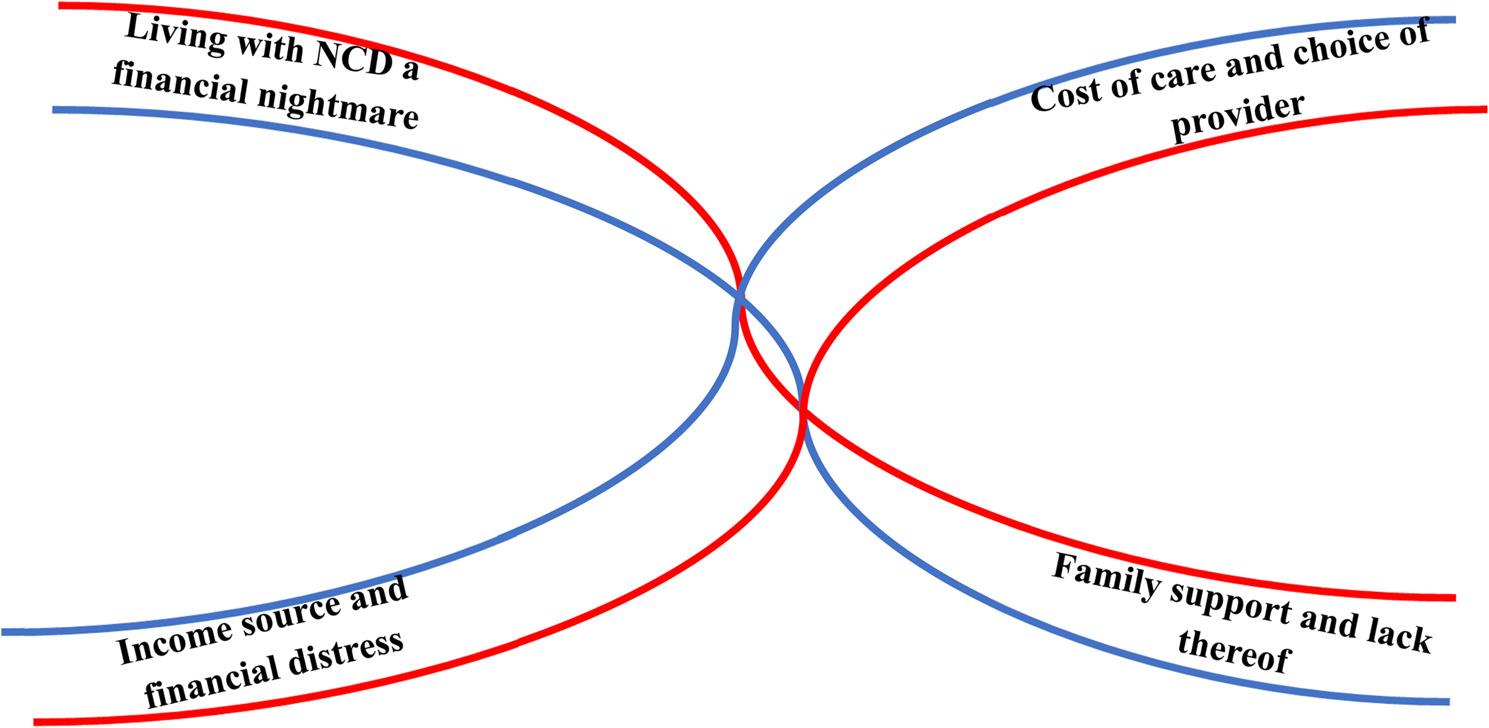
Overview of qualitative themes representing lived experiences of older adults living with NCDs. NCD= Non-Communicable Diseases

The qualitative interviews revealed four interconnected themes reflecting the lived experiences of older adults regarding the financial impact of NCDs. These themes explore the financial burden of NCDs, the link between income sources and financial distress, the influence of treatment costs on healthcare provider selection, and the role of family support in navigating these challenges (Fig. 3).

### Theme 1: living with NCD is a financial nightmare

Participants reported living with NCD as a financial nightmare. Screening, diagnosis, treatment and follow up required frequent expenditures most of which the households were not prepared for. Recurrent tests, procedures, and travel associated with healthcare seeking contributed to high cost of care. One participant with chronic kidney disease who was on dialysis reported her situation as follows.

*“We are paying around 500 rupees towards transport for one episode of dialysis. If we pay 500 for one dialysis; for one week*,* we have to do 2 dialysis*,* …… In a month there are four weeks*,* that mean we are doing 8 dialysis in a month. Approximately for transport alone we are spending around 4000 per month” (P-22*,* 63Y*,* Female*,* HT & CKD & Asthma)*.

Further, individuals also reported that an NCD diagnosis like Cancer, requiring critical care impoverishes the household unless they are supported by an alternate payment mechanism. One participant reported

*“For treatment in hospital alone we spent about Rs.27 lakhs…we had some money in our hand…my relatives*,* her (wife) sister brought money and gave to us in hospital…we mortgaged our jewels and repaid it and took loan….my wife had government insurance as part of her job it reduced the cost by around 4.25 to 4.5 lakhs.” (P-26*,* 60Y*,* Male*,* Cancer*,* DM & HT)*.

### Theme 2: income source and financial distress

The income source impacted if or not the households went into financial distress when seeking care. Households with a formal income source such as a salary or a pension were better prepared to handle the expenses associated with NCDs.

*“We didn’t spend much money for the operation*,* 5 lakh rupees was credited through the card (employer insurance card)… money was not a big problem as my husband earns pension.” (P-1*,* 63Y*,* Female*,* DM*,* Cholesterol*,* CHD)*.

In other end, poor households and those without a formal source of income or a payment mechanism like an insurance resorted to borrowing or selling assets to finance healthcare. These are the households which are mostly poor, were eligible or receiving government supported subsidies/benefits but still were pushed to financial distress.

*“All (healthcare expenses) was done by borrowing the money only…. We had to sell our land to repay all the debts and borrowed money.” (P-18*,* 60Y*,* Male*,* DM& Injury)*.

### Theme 3: cost of care and choice of provider

The choice of provider (i.e., public or private) was dependent on the household’s ability to pay. While public healthcare facilities were preferred by individuals with limited resources, some participants were observed to shift their provider from private to public as an adjustment to high cost of managing NCDs, whereas several even considered forgoing care.

*“If we have 300 [rupees]*,* we go to clinic and see the doctor there*,* if we have around 1000 [rupees]*,* we go to hospital and get treatment there” (P-7*,* 65Y*,* Male*,* HT & Renal calculi).*

*“Now these two years only I am taking the medicines from the government. We are staying at home only*,* how can we go out and pay money to get the medicine? that is not possible right” (P-19*,* 60 + Y*,* Female*,* DM & HT)*.

### Theme 4: family support and lack thereof

For older adults living with NCDs, family support facilitated disease management by improving healthcare access, providing care and paying for healthcare

*“The remaining one lakh rupees we took care of as I said my children spent a portion*,* they are working*,* my pension and my wife pension is there as well.” (P-11*,* 70Y*,* Male*,* DM & Injury)*.

Lack of family support on other hand curtailed access to healthcare, particularly among older adults with NCD multimorbidity.

*“When my children are not coming to take me to hospital*,* I will have to wait till they come without going to the hospital*,* what else can I do (…) Who else will take us.” (P-17*,* 80Y*,* Female*,* HT & multiple injuries).*

## Discussion

We investigated the household economic condition, catastrophic health expenditure, and impoverishment among older adult households with NCDs. Four in every ten households incurred CHE and a third of households were impoverished due to health care costs. Our estimates for CHE and impoverishment are higher than the recent estimates based the national sample surveys, which reported the incidence of CHE of around 29%, and impoverishment of 12.2% [30]. The difference is largely attributable to non-communicable disease status of our study sample [7, 9]. Further, > 16% of the households reported household economic condition worsened compared to previous years, which can be attributed to recurrent nature of NCD care. Participants in qualitative interviews reported recurrent costs associated with seeking healthcare for NCDs had a major financial impact. Treatment for procedures like haemodialysis were documented to incur an average monthly health related out of pocket expenditure of INR 16,672/- [31]. Our quantitative and qualitative findings together indicate that the cost of recurrent healthcare for NCDs further escalates with distance, as costs involved in transportation increase the risk of CHE among older adult households in rural areas [29]. Further, financing the healthcare and treatment seeking process through loans and sale of assets as observed in our interviews, is known to result in health shocks chronically destabilizing the economic status of the household (Table 4) [32]. 

**Table 4:** Joint display highlighting the convergence of quantitative and qualitative findings

Quantitative findings	Qualitative findings
Financial impact of living with NCDs CHE: 41.2%(40.4–42.0) Impoverishment: 28.4% (27.7–29.2) Worsened HH economic condition: 16.5% (16.0–17.0)	“All (healthcare expenses) was done by borrowing the money only…. We had to sell our land to repay all the debts and borrowed money.” (*P*-18, 60Y, Male, DM& Injury)
Rural residence ^a^: HH economic condition worsened-(AOR = 2.35[2.11–2.62]); CHE-(AOR = 2.01[1.85–2.18]); Impoverishment-(AOR = 4.38[3.91–4.90]) OP Care ^b^: HH economic condition worsened- (AOR = 1.39[1.23–1.58]); IP & OP Care ^c^: CHE-(AOR = 3.31[3.05–3.59]); Impoverishment-(AOR = 1.37[1.23–1.52])	*“We are paying around 500 rupees towards transport for one episode of dialysis. If we pay 500 for one dialysis; for one week*,* we have to do 2 dialysis …… In a month there are four weeks*,* that mean we are doing 8 dialysis in a month. Approximately for transport alone we are spending around 4000 per month” (P-22*,* 63Y*,* Female*,* HT & CKD & Asthma)*
Poor households ^d^: HH economic condition worsened-(AOR = 2.51[2.18–2.89]); CHE-(AOR = 1.14[1.06–1.24]; Impoverishment-(AOR = 22.78[20.19–25.70]) Government subsidies & Benefits ^e^: HH economic condition worsened-(AOR = 2.51[2.18–2.89]); CHE-(AOR = 3.26[2.84–3.75]) Government Health Insurance ^f^: Impoverishment- (AOR = 1.43[1.06–1.92])	*“We didn’t spend much money for the operation*,* 5 lakh rupees was credited through the card (employer insurance card)… money was not a big problem as my husband earns pension.” (P-1*,* 63Y*,* Female*,* DM*,* Cholesterol*,* CHD)* *“If we have 300*,* we go to clinic and see the doctor there*,* if we have around 1000*,* we go to hospital and get treatment there” (P-7*,* 65Y*,* Male*,* HT & Renal calculi).*

NCD = Non communicable diseases; HH= Household; CHE= Catastrophic Health Expenditure; AOR= Adjusted Odds Ratios; a= response category of the independent variable “place of residence” with ‘urban residence’ as a reference category; b= response category of the independent variable “type of care” with ‘no care’ as a reference category; c = response category of the independent variable “type of care” with ‘OP care’ as a reference category; d=response category of the independent variable “Wealth Index” with ‘rich’ as a reference category; e= response category of the independent variable “income source” with ‘individual salary’ as a reference category; f = response category of the independent variable “Health Insurance” with ‘private health insurance’ as a reference category: Note-The AOR reported were derived through logistic regression analysis, details results are provided in Tables 2 and 3

The financial impact of NCDs and associated healthcare use was predominantly felt among most vulnerable households. Female headed households, poor households, rural residents, and those with agriculture or government subsidies as major income sources had higher odds of catastrophic health expenditures, impoverishment, and a worsened economic status. While evidence point that female-headed households have a higher probability to be enrolled into a PFHI schemes [33], our findings indicate that they also have a higher probability of impoverishment. A greater vulnerability to health shocks among female-headed households have been previously reported in Indian settings [34]. This could be largely attributed to vulnerabilities of low education, limited access to resources and credit, poverty, low income and other social determinants which intersect with the gender of household head [34, 35]. Similar to previous studies, we also observed the highest proportion of worsening economic condition, CHE and impoverishment among scheduled caste households, compared to other social groups [30]. In concurrence with our findings, earlier nationally representative surveys report that poor and poorest households faced significantly more CHE and impoverishment despite having lower healthcare expenses compared to their richer counter parts [30, 36]. Further, between 2014 and 2018, the percentage of households pushed into poverty due to healthcare expenses attributable to NCDs raised from 8.5% to 12.4%, and 78.7% of already poor households further deepening into poverty [36]. In extension to the role of income and poverty, our qualitative findings reveal that participants actively chose healthcare providers based on their financial capacity, often resulting in forgone care or undertreatment due to economic constraints (Table 4).

The public funded health insurance (PFHI) programmes were launched to protect the poor and vulnerable households from catastrophic healthcare expenditures [37]. Our findings indicate that, while those with a government financed health insurance did not differ significantly from private health insurance covered households in terms of incurring CHE, they were more likely to be impoverished similar to that of non-covered households. Although our qualitative analysis did not compare public and private insurance plans, the participants with an ‘employer supported insurance’ reported financing healthcare not to be challenging, while those without one reported to borrow or sell assets to pay for healthcare (Table 4). Together, our findings point towards a limited effectiveness of government funded health insurance programmes in ensuring financial protection as previously documented [13, 37–40], more specifically in context of older adults living with NCDs. A higher preference of private providers for NCD treatment [15, 41], limited coverage and operational challenges with public funded schemes [13, 37, 38, 40], explain our findings. However, studies with sufficient granularity are needed to further explain if the poor protection of PFHI schemes could be attributed to limited coverage scope, utilization patterns, or provider behaviours.

Our findings also highlight on the nature of healthcare requirement for NCDs amplifying their financial impact. The NCD multimorbidity being associated with worsening economic condition and catastrophic health expenditures concurs with the existing evidence [9]. NCD multimorbidity increases the number of outpatient visits, inpatient hospitalizations, and additional morbidity increasing the cost of care [9, 36, 42]. Long term NCD care involves a progressive use of inpatient and outpatient care [2, 7, 10], whereas within their limitations, most of PFHI schemes focus on secondary and tertiary care [37]. Also, the risk of catastrophic health expenditures correlates with high out of pocket expenditures for medications (Supplementary file 1, item S4) [7, 43], which remain a critical bottleneck in public healthcare delivery systems.

Our findings expand on the previous studies based on National Sample Survey and LASI indicating that the presence of NCDs in older adult households exacerbates health inequities resulting in catastrophic health expenditures, impoverishment, and worsening household economic condition over a long period of time. The financial burden of managing NCDs was particularly severe among vulnerable households, while households relying on formal employer-sponsored insurance, salaries, pensions, or family support experienced relatively better protection. In contrast, most others were compelled to sell assets or incur debt to finance treatment. Existing safety nets, such as PFHI schemes, proved largely ineffective in shielding households from impoverishment.

In India, safeguarding older adult households from financial catastrophe require a multi-pronged approach that expands access to NCD care through strengthened primary healthcare services, with an emphasis on early screening, prevention, and management of multi-morbidity. Strengthening health and wellness centres, capacity building of grassroots healthcare providers, and improving access to secondary and tertiary healthcare in public sector is crucial to protect low-income older adult households, rural residents, and those from vulnerable social groups. Leveraging community health workers to implement programmes akin to “Makkalai Thedi Maruthuvam” could be useful. Further, ensuring affordable access to medicines, diagnostics, and subsidised transport to healthcare facilities could protect the most vulnerable. PFHI schemes need more robust implementation, with improvements in coverage, service provision, empanelment of facilities, and simplified claims processes.

### Limitations

Our research relied on self-reported survey data and may be subject to recall bias. Also, our research does not adequately account for the impact of more comprehensive insurance programmes like Pradhan Mantri Jan Arogya Yojana, which was implemented during the period of LASI survey. Owing to logistical constrains, our qualitative field work was limited only to Tamil Nadu, which may impact the generalizability of our qualitative findings to older adults in other Indian states.

## Supplementary Information

Supplementary Material 1.

Supplementary Material 2.

Supplementary Material 3.

## Data Availability

The data used for the quantitative phase of this study is accessible for researchers by submitting a formal request on the website of the International Institute for Population Sciences (IIPS), Mumbai ( [https://www.iipsdata.ac.in/datacatalog\_detail/5](https:/www.iipsdata.ac.in/datacatalog_detail/5) ). The anonymised data from the qualitative interviews are available from the corresponding author on reasonable request.

## Abbreviations

AOR: Adjusted odds ratio
CGHS: Central government health scheme
CHD: Coronary heart diseases
CHE: Catastrophic health expenditure
CKD: Chronic Kidney disorder
CTP: Capacity to pay
DM: Diabetes mellitus
HT: Hypertension
IIPS: International Institute for Population Sciences
IP: In patient
LASI: Longitudinal aging study in India
NCD: Non–communicable diseases
OP: Out patient
PFHI: Public funded health insurance
