# Supplementary material for: Financial impact of non-communicable diseases on households with older adults in india: a mixed methods study
Source: BMC Geriatr. 2026 Apr 18;26:772. doi: 10.1186/s12877-026-07493-9 (PMC13224535; doi:10.1186/s12877-026-07493-9)

**Supplementary file 1**

**Item S1**: Conceptual framework outlining the factors associated with financial impact of NCDs among older adult households with NCDs

**Financial impact of NCDs**

Worsening household economic condition

Catastrophic Health Expenditure

Impoverishment

**Healthcare use**

**No**

**Yes**

-Inpatient

-Out Patient

-Inpatient and Outpatient Care

Socio-demographic Characteristics

NCD Status

Economic Status

Health Insurance Coverage

**Outcome variables**

**Predictor variables**

NCD: Non communicable Diseases; Socio-demographic characteristics include sex of the household head, caste of the household head, education of household head and income source.

**Item S2: Outcome Variables**

**Calculation of Catastrophic Health Expenditure and Impoverishment**

| **Variables**  **(Outcome)** | **Description/Questions used in the LASI questionnaire** | **Computation of response categories** |
| --- | --- | --- |
| Outpatient health expenditure  (In last 1 year) | In past 30 days how much did your household spend on  Medication, Tests (blood test, urine test, X-ray, ECG, etc) during outpatient visits, Doctors fees (for non-institutional, outpatient services), any other medical expense, total healthcare expenses in last 30 days.  (CO108-CO112) | (CO108+ CO109+ CO110+ CO111+ CO112) * 12 |
| Inpatient health expenditure  (In last 1 year) | In past 12 months, how much did your household spend on Hospitalization and nursing home stays, Tests (X-ray, ECG, USG, CT scan, MRI, blood test, urine test, etc), Medicine, Doctors fees, any other medical expenses you paid during hospitalization, what were your total household health expenses on inpatient visits in past 12 months.  (CO202-CO207) | (CO202+ CO203+ CO204+ CO205+ CO206+ CO207) |
| Total heath expenditure  (In last 1 year) | Sum of outpatient health expenditure and inpatient health expenditure. | Outpatient health expenditure+ Inpatient health expenditure |
| Food expenditure  (In last 1 year) | In last 7 days, what was the total market value of your household’s consumption on cereals and cereal products, pulses and pulse products, edible oil, salt and spices, milk and milk products; sugar and sugar products, fruits and vegetables, eggs, chicken, meat, fish or any other non-vegetarian food item, non-alcoholic drinks and beverages, alcoholic drinks, all other food items, food eaten outside home.  (CO002a-CO002j) | (CO002a+ CO002b+ CO002c+ CO002d+ CO002e+ CO002f+ CO002g+ CO002h+ CO002i+ CO002j) * 52 |
| Non-food expenditure  (In last 1 year) | In past 30 days, how much did your household spend on communication fees, fuels and utilities such as gas, house rent, tobacco and tobacco products, personal toiletries, entertainment, other expenses.  (CO101-CO107) | (CO101+ CO102+ CO103+ CO104+ CO105+ CO106+ CO107) * 12 |
| Other expenditures  (In last 1 year) | In past 12 months, how much did your household spend on clothing, bedding, linens, footwear, education and training, durables, jewelry and ornaments, rituals, festivals and ceremonies, taxes and non-health insurance premium, loan repayment, any other expenses.  (CO209-CO216) | CO209+ CO210+  CO211+ CO212+ CO213+ CO214+ CO215+ CO216 |
| Total household expenditure  (In last 1 year) | Sum of food expenditure, non-food expenditure and other expenditures. | Food expenditure+ non-food expenditure+ other expenditures |

**NOTE:** COxxx represent the variable ID in LASI survey data set

**Calculating Catastrophic Health Expenditure and Impoverishment**

***Notes: OOPCTP – Out of pocket to capacity to pay ratio, OOPE – out of pocket expenditure***

Catastrophic health expenditure(CHE) is calculated following the capacity to pay(CTP) approach. The steps involve calculation total household expenditure and food expenditure and subsequent share of food expenditure in total household expenditure. The equivalent household size calculated by raising the household size to power 0.56. The equivalent food expenditure is calculated by division of household food expenditure by equivalent household size. Poverty line is determined by the average of median equivalent food expenditure of all households. The subsistence expenditure is arrived by multiplying the poverty line value by equivalent household size. The CTP is determined by two ways, if subsistence expenditure is less than or equal to food expenditure the difference between the total household expenditure and subsistence expenditure is considered as CTP. Else the difference between total household expenditure and food expenditure is considered as CTP. If the out-of-pocket expenditure (OOPE) exceeds 40% of the CTP it is considered as CHE.

While for impoverishment if the total household expenditure is greater than or equal to subsistence expenditure and household expenditure excluding OOPE is less than subsistence spending the household is considered as impoverished. Also, poor households incurring health expenditure is also considered impoverished.

**Household economic condition compared to previous years**

| **Variables**  **(Outcome)** | **Description/Questions used in the LASI questionnaire** | **Response/recoded categories** |
| --- | --- | --- |
| Household Economic condition compared to previous year | Would you say your household’s overall economic condition has improved, stayed about the same, or worsened, compared to two years ago? (IN902) | 1 – Improved  2 – Same  3 – Worsened |

**NOTE:** IN902 represent the variable ID in LASI survey data set

**Item S3: Computation of Predictor Variables**

| **Variables**  **(Predictor)** | **Description/Questions used in the LASI questionnaire** | **Response/recoded categories** |
| --- | --- | --- |
| Hypertension | Has any health professional ever told you that you have hypertension or high blood pressure (HT002) | 0 – No  1 – Yes |
| Diabetes | Has any health professional ever told you that you have diabetes or high blood sugar (HT003) | 1 – Yes  0 – No |
| Cancer | Has any health professional ever told you that you have cancer or malignant tumor (HT004) | 0 – No  1 – Yes |
| Chronic lung diseases | Has any health professional ever told you that you have chronic lung disease such as asthma, chronic obstructive pulmonary disease/ chronic bronchitis or other chronic lung problems (HT005) | 0 – No  1 – Yes |
| Chronic heart diseases | Has any health professional ever told you that you have chronic heart disease such as coronary heart disease (heart attack or myocardial infarction), congestive heart failure, or other chronic heart problems (HT006) | 0 – No  1 – Yes |
| Stroke | Has any health professional ever told you that you have stroke (HT007) | 0 – No  1 – Yes |
| Arthritis | Has any health professional ever told you that you have arthritis or rheumatism, osteoporosis or other bob/joint diseases (HT008) | 0 – No  1 – Yes |
| Neurological problems | Has any health professional ever told you that you have any neurological, or psychiatric problems such as depression, Alzheimer’s/dementia, unipolar/bipolar disorders, convulsion, Parkinson’s etc (HT009) | 0 – No  1 – Yes |
| Cholesterol | Has any health professional ever told you that you have high cholesterol (HT010) | 0 – No  1 – Yes |
| Other chronic conditions | Have you been diagnosed with any of the following chronic conditions or diseases. (HT011) | 0 – No  1 – Yes |
| **Non-communicable disease status** | Sum of responses to questions regarding presence of chronic diseases/conditions. (i.e., HT002+ HT003+ HT004+ HT005+ HT006+ HT007+ HT008+ HT009+ HT010+ HT011) | 0 – No  1 – Single NCD (yes to any one of the above diseases)  2 – NCD multimorbidity (yes for more than one of the above diseases) |

(LASI – Longitudinal Ageing Survey of India)

| **Variable (Household characteristics)** | **Description/Question in the LASI questionnaire.** | **Response/ recoded categories** |
| --- | --- | --- |
| Residence | Place of residence | 1 – Urban  2 – Rural |
| Epidemiological transition level(ETL) group | State of respondent  Low ETL – Bihar, Jharkhand, Uttar Pradesh, Rajasthan, Meghalaya, Assam, Chhattisgarh, Madhya Pradesh and Odisha  Lower Middle ETL – Arunachal Pradesh, Mizoram, Nagaland, Uttarakhand, Gujarat, Tripura and Manipur.  Higher Middle ETL – Haryana, Delhi, Telangana, Andra Pradesh, Jammu and Kashmir, Karnataka, West Bengal, Maharashtra and Union territories other than Delhi.  High ETL – Himachal Pradesh, Punjab, Tamil Nadu, Goa and Kerala. | 1 – Low ETL  2 – Lower Middle ETL  3 – Higher Middle ETL  4 – High ETL |
| Caste of the household head | What is household head’s caste or tribe? (CV016) | 1 – OBC  2 – Others  3 – SC  4 – ST |
| Wealth Index | MPCE quintile | 1 – Richest  2 – Richer  3 – Middle  4 – Poorer  5 – Poorest |
| Educational status of household head | What is the highest level of education completed? (CV010) | 0 – not attended school  1 – up to middle school  2 – up to diploma/higher secondary  3 – graduate and above  9999 – don’t know |
| Insurance coverage | Does any member in your household have…?  HI002a – Central Government Health Scheme  HI002b – Employees State Insurance Scheme  HI002c – Rashtriya Swasthya Bima Yojana  HI002d – Other central government health insurance schemes  HI002e – State government health insurance schemes  HI002f – community/ cooperative health insurance schemes  HI002g – Medical reimbursement from an employer  HI002h – Health insurance through an employer  HI002i – privately purchased commercial health insurance  HI002j – Others | 1 – Government  2 – Private  3 – not covered |
| Income source of Household | We would like to ask you some questions about the income of your household.  Agricultural _income  Non-agricultural _business _income  Wages _salary _income  Pension _income  Government _transfers  Other _HH _income  (Based on highest contributor to the total household income the major income source of the household is determined) | 1 – Agricultural & Non-agricultural business  2 – Individual income  3 – Government subsidies & other income |

(LASI – Longitudinal Ageing Survey of India)

**Item S4:** Spectrum of healthcare expenditures among households by occurrence of CHE


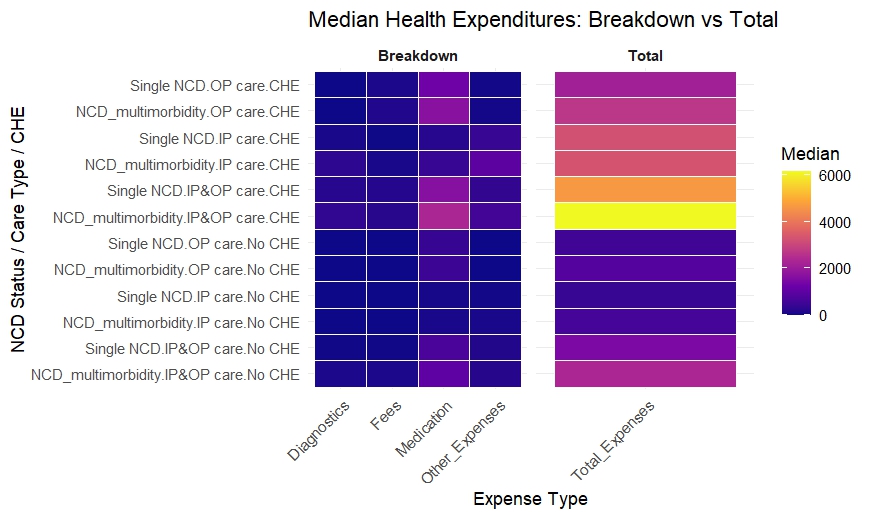

Supplement: Supplementary file 1 — Supplementary Material 1. [file 12877_2026_7493_MOESM1_ESM.docx]
