# Supplementary material for: Financial impact of non-communicable diseases on households with older adults in india: a mixed methods study
Source: BMC Geriatr. 2026 Apr 18;26:772. doi: 10.1186/s12877-026-07493-9 (PMC13224535; doi:10.1186/s12877-026-07493-9)
Supplement: Supplementary file 2 — Supplementary Material 2. [file 12877_2026_7493_MOESM2_ESM.docx]

**Supplementary file 3: Gender and Rural/urban stratification of CHE, impoverishment and household economic condition.**

Table 1 : Characteristics of older adult households studied (n = 14067)

| **CHARACTERISTICS** | **Rural (%)**  **(n = 8367)** | **Urban (%)**  **(n = 5700)** |
| --- | --- | --- |
| **Sex of household head** |  |  |
| Male | 82.4 | 77.1 |
| Female | 17.6 | 22.9 |
| **Caste of household head** |  |  |
| Other backward class | 45.4 | 46.6 |
| Scheduled caste | 22.5 | 11.1 |
| Scheduled tribe | 7.2 | 3.0 |
| Others^a^ | 24.9 | 39.3 |
| **Education of household head** |  |  |
| No schooling | 41.8 | 18.3 |
| Up to middle school | 38.6 | 34.1 |
| Up to diploma/higher secondary | 14.8 | 29.3 |
| Graduate and above | 4.4 | 17.4 |
| **Income source** |  |  |
| Agriculture & Non-agricultural business | 46.6 | 23.2 |
| Individual salary | 39.4 | 65.5 |
| Government subsidies and other income | 14.0 | 11.3 |
| **Wealth index** |  |  |
| Poor | 38.7 | 39.2 |
| Middle | 20.9 | 21.9 |
| Rich | 40.4 | 38.9 |
| **Epidemiological transition level** |  |  |
| Low ETL | 41.4 | 22.3 |
| Lower middle ETL | 5.1 | 8.4 |
| Higher middle ETL | 39.3 | 52.6 |
| High ETL | 14.2 | 16.8 |
| **Health Insurance coverage** |  |  |
| Government | 24.6 | 17.6 |
| Private | 2.3 | 6.2 |
| Not covered | 73.1 | 76.2 |
| **NCD status** |  |  |
| Single NCD | 57.2 | 43.5 |
| NCD multimorbidity | 42.8 | 56.5 |
| **Type of care** |  |  |
| OP care only | 58.4 | 52.3 |
| IP care only | 3.6 | 4.5 |
| Both OP & IP care | 19.9 | 18.1 |
| Care not availed | 18.1 | 25.1 |

ETL: Epidemiological Transition Level; **Low ETL**- Bihar, Jarkhand, Uttar Pradesh, Rajasthan, Meghalaya, Assam, Chhattisgarh, Madhya Pradesh, Odisha; **Lower Middle ETL**- Arunachal Pradesh, Mizoram, Nagaland, Uttarakahnd, Gujarat, Tripura, Manipur; **Higher Middle ETL**- Haryana, Delhi, Telangana, Andhra Pradesh, Jammu and Kashmir, Karnataka, West Bengal, Maharashtra, and Union territories other than Delhi; **High ETL**- Himachal Pradesh, Punjab, Tamil Nadu, Goa, and Kerala; NCD: Non-Communicable Diseases. OP: Outpatient care, IP: Inpatient care; ^a^Others included the caste groups which were not reported to be scheduled castes, scheduled tribes or other backward castes. The self-reported diagnosis with hypertension, diabetes, cancer, chronic lung diseases, chronic heart diseases, stroke, arthritis, neurological problems, high cholesterol or other chronic conditions were included in assessment of NCD status. Note: The percentages reported are weighted percentages

Table 2 : Characteristics of older adult households studies (n = 14067)

| **CHARACTERISTICS** | **Female (%)**  **(n = 2957)** | **Male(%)**  **(n = 11110)** |
| --- | --- | --- |
| **Caste of household head** |  |  |
| Other backward class | 46.4 | 45.7 |
| Scheduled caste | 19.9 | 18.2 |
| Scheduled tribe | 5.2 | 5.8 |
| Others^a^ | 28.5 | 30.4 |
| **Education of household head** |  |  |
| No schooling | 60.7 | 26.9 |
| Up to middle school | 27.2 | 39.4 |
| Up to diploma/higher secondary | 9.1 | 22.6 |
| Graduate and above | 2.7 | 10.5 |
| **Income source** |  |  |
| Agriculture & Non-agricultural business | 30.4 | 40.2 |
| Individual salary | 48.3 | 48.7 |
| Government subsidies and other income | 21.3 | 11.1 |
| **Wealth index** |  |  |
| Poor | 39.8 | 38.6 |
| Middle | 19.6 | 21.7 |
| Rich | 40.6 | 39.7 |
| **Residence** |  |  |
| Urban | 41.5 | 33.8 |
| Rural | 58.5 | 66.2 |
| **Epidemiological transition level (ETL)** |  |  |
| Low ETL | 24.9 | 37.0 |
| Lower middle ETL | 6.9 | 6.1 |
| Higher middle ETL | 47.3 | 43.2 |
| High ETL | 20.8 | 13.7 |
| **Health Insurance coverage** |  |  |
| Government | 22.7 | 22.0 |
| Private | 3.1 | 3.8 |
| Not covered | 74.3 | 74.1 |
| **NCD status** |  |  |
| Single NCD | 54.5 | 51.8 |
| NCD multimorbidity | 45.5 | 48.2 |
| **Type of care** |  |  |
| OP care only | 50.8 | 57.6 |
| IP care only | 5.6 | 3.5 |
| Both OP & IP care | 18.0 | 19.6 |
| Care not availed | 25.7 | 19.3 |

ETL: Epidemiological Transition Level; **Low ETL**- Bihar, Jarkhand, Uttar Pradesh, Rajasthan, Meghalaya, Assam, Chhattisgarh, Madhya Pradesh, Odisha; **Lower Middle ETL**- Arunachal Pradesh, Mizoram, Nagaland, Uttarakahnd, Gujarat, Tripura, Manipur; **Higher Middle ETL**- Haryana, Delhi, Telangana, Andhra Pradesh, Jammu and Kashmir, Karnataka, West Bengal, Maharashtra, and Union territories other than Delhi; **High ETL**- Himachal Pradesh, Punjab, Tamil Nadu, Goa, and Kerala; NCD: Non-Communicable Diseases. OP: Outpatient care, IP: Inpatient care; ^a^Others included the caste groups which were not reported to be scheduled castes, scheduled tribes or other backward castes. The self-reported diagnosis with hypertension, diabetes, cancer, chronic lung diseases, chronic heart diseases, stroke, arthritis, neurological problems, high cholesterol or other chronic conditions were included in assessment of NCD status. Note: The percentages reported are weighted percentages

Table 3 : Factors associated with CHE at urban and rural (n =10963)

| **Variables** | **Rural**  **(n = 6698)** | **CHE** | **Urban (n = 4265)** | **CHE** |
| --- | --- | --- | --- | --- |
|  | **%** | **AOR(95% CI)** | **%** | **AOR (95% CI)** |
| **Gender of household head** |  |  |  |  |
| Female | 49.9 | 1.23 (1.09 - 1.38)* | 35.4 | 1.18 (1.01 - 1.37)* |
| Male (Ref) | 45.8 |  | 28.4 |  |
| **Caste** |  |  |  |  |
| Scheduled caste | 49.0 | 1.10 (0.99 - 1.23) | 28.8 | 0.97 (0.77 - 1.21) |
| Scheduled tribe | 36.8 | 0.63 (0.53 - 0.75)** | 48.2 | 1.33 (0.89 – 2.00) |
| Others | 45.0 | 0.88 (0.79 - 0.97)* | 29.3 | 1.13 (0.98 - 1.31) |
| Other backward class (Ref) | 47.5 |  | 29.9 |  |
| **Income source** |  |  |  |  |
| Agricultural & non-agricultural business | 47.8 | 1.19 (1.09 - 1.30)** | 32.2 | 1.35 (1.15 - 1.57)** |
| Government subsidies & others | 53.1 | 1.59 (1.39 - 1.81)** | 45.8 | 2.25 (1.80 - 2.80)** |
| Individual salary (Ref) | 42.7 |  | 27.1 |  |
| **Wealth index** |  |  |  |  |
| Middle | 45.8 | 0.98 (0.88 - 1.09) | 27.1 | 1.03 (0.85 - 1.24) |
| Poor | 47.0 | 1.13 (1.03 - 1.25)* | 29.5 | 1.17 (1.01 - 1.37)* |
| Rich (Ref) | 46.4 |  | 31.7 |  |
| **Epidemiological transition level** |  |  |  |  |
| Low ETL | 48.2 | 1.64 (1.42 - 1.88)** | 27.1 | 0.9 (0.73 - 1.12) |
| Lower middle ETL | 27.8 | 0.67 (0.53 - 0.86)* | 19.1 | 0.53 (0.40 - 0.72)** |
| Higher middle ETL | 49.1 | 1.64 (1.43 - 1.89)** | 32.9 | 0.95 (0.78 - 1.15) |
| High ETL (Ref) | 38.2 |  | 31.9 |  |
| **Health insurance** |  |  |  |  |
| Government | 42.6 | 1.25 (0.94 - 1.66) | 29.5 | 1.32 (0.97 - 1.78) |
| Not covered | 48.0 | 1.43 (1.09 - 1.88)* | 30.5 | 1.28 (0.98 - 1.68) |
| Private(Ref) | 37.0 |  | 26.7 |  |
| **NCD status** |  |  |  |  |
| NCD multimorbidity | 50.2 | 1.32 (1.21 - 1.43)** | 32.5 | 1.22 (1.06 - 1.40)* |
| Single NCD (Ref) | 43.5 |  | 26.5 |  |
| **Type of care** |  |  |  |  |
| IP care | 38.9 | 1.00 (0.81 - 1.23) | 43.8 | 3.22 (2.50 - 4.14)** |
| IP & OP care | 64.2 | 2.79 (2.53 - 3.09)** | 55.5 | 4.93 (4.25 - 5.73)** |
| OP care (Ref) | 40.9 |  | 20.1 |  |

**Dependent Variables:** Catastrophic Health Expenditure (CHE)= No (Ref), Yes;

ETL: Epidemiological Transition Level; **Low ETL**- Bihar, Jarkhand, Uttar Pradesh, Rajasthan, Meghalaya, Assam, Chhattisgarh, Madhya Pradesh, Odisha; **Lower Middle ETL**- Arunachal Pradesh, Mizoram, Nagaland, Uttarakahnd, Gujarat, Tripura, Manipur; **Higher Middle ETL**- Haryana, Delhi, Telangana, Andhra Pradesh, Jammu and Kashmir, Karnataka, West Bengal, Maharashtra, and Union territories other than Delhi; **High ETL**- Himachal Pradesh, Punjab, Tamil Nadu, Goa, and Kerala; HH=Household, IP= Inpatient, OP=Out Patient NCD: Non-Communicable Diseases, Ref= reference category.

The self-reported diagnosis with hypertension, diabetes, cancer, chronic lung diseases, chronic heart diseases, stroke, arthritis, neurological problems, high cholesterol or other chronic conditions were included in assessment of NCD status. * - p-value <0.05, **- p-value <0.001. Note: The percentages reported are weighted percentages

Table 4 : Factors associated with impoverishment at urban and rural (n =10963)

| **Variables** | **Rural (n = 6698)** | **Impoverishment** | **Urban (n = 4265)** | **Impoverishment** |
| --- | --- | --- | --- | --- |
|  | **%** | **AOR(95% CI)** | **%** | **AOR (95% CI)** |
| **Gender of household head** |  |  |  |  |
| Female | 48.3% | 2.34 (2.04 - 2.69)** | 15.6% | 1.39 (1.11 - 1.73)* |
| Male (Ref) | 32.4% |  | 13.6% |  |
| **Caste** |  |  |  |  |
| Scheduled caste | 42.0% | 1.19 (1.05 - 1.36)* | 22.1% | 1.14 (0.87 - 1.49) |
| Scheduled tribe | 42.1% | 1.18 (0.96 - 1.45) | 4.3% | 0.40 (0.16 - 0.98)* |
| Others | 26.6% | 0.85 (0.74 - 0.97)* | 11.4% | 0.80 (0.65 - 0.99)* |
| Other backward class (Ref) | 35.0% |  | 15.4% |  |
| **Income source** |  |  |  |  |
| Agricultural & non-agricultural business | 34.8% | 1.59 (1.42 - 1.78)** | 16.2% | 1.41 (1.14 - 1.75)* |
| Government subsidies & others | 51.0% | 3.47 (2.95 - 4.07)** | 23.3% | 2.48 (1.85 - 3.31)** |
| Individual salary (Ref) | 29.5% |  | 12.1% |  |
| **Wealth index** |  |  |  |  |
| Middle | 28.9% | 3.47 (3.01 - 3.99)** | 5.6% | 4.37 (2.75 - 6.94)** |
| Poor | 68.4% | 21.1 (18.49 - 24.07)** | 33.7% | 38.82 (26.64 - 56.55)** |
| Rich (Ref) | 10.8% |  | 1.4% |  |
| **Epidemiological transition level** |  |  |  |  |
| Low ETL | 39.4% | 1.38 (1.15 - 1.65)** | 16.9% | 0.81 (0.61 - 1.08) |
| Lower middle ETL | 22.6% | 0.93 (0.68 - 1.28) | 5.7% | 0.32 (0.19 - 0.52)** |
| Higher middle ETL | 33.5% | 1.35 (1.12 - 1.62)* | 13.6% | 1.01 (0.77 - 1.33) |
| High ETL (Ref) | 26.7% |  | 16.0% |  |
| **Health insurance** |  |  |  |  |
| Government | 33.3% | 1.32 (0.91 - 1.9) | 14.1% | 1.38 (0.83 - 2.3) |
| Not covered | 35.7% | 1.24 (0.87 - 1.78) | 14.7% | 1.53 (0.96 - 2.45) |
| Private(Ref) | 24.0% |  | 7.3% |  |
| **NCD status** |  |  |  |  |
| NCD multimorbidity | 31.7% | 0.99 (0.89 - 1.1) | 12.0% | 0.87 (0.72 - 1.05) |
| Single NCD (Ref) | 37.4% |  | 17.2% |  |
| **Type of care** |  |  |  |  |
| IP care | 37.6% | 1.21 (0.93 - 1.56) | 13.5% | 0.98 (0.65 - 1.47) |
| IP & OP care | 30.0% | 1.28 (1.13 - 1.45)** | 15.0% | 1.72 (1.38 - 2.15)** |
| OP care (Ref) | 36.4% |  | 13.9% |  |

**Dependent Variables:** Impoverishment=No (Ref), Yes.

ETL: Epidemiological Transition Level; **Low ETL**- Bihar, Jarkhand, Uttar Pradesh, Rajasthan, Meghalaya, Assam, Chhattisgarh, Madhya Pradesh, Odisha; **Lower Middle ETL**- Arunachal Pradesh, Mizoram, Nagaland, Uttarakahnd, Gujarat, Tripura, Manipur; **Higher Middle ETL**- Haryana, Delhi, Telangana, Andhra Pradesh, Jammu and Kashmir, Karnataka, West Bengal, Maharashtra, and Union territories other than Delhi; **High ETL**- Himachal Pradesh, Punjab, Tamil Nadu, Goa, and Kerala; HH=Household, IP= Inpatient, OP=Out Patient NCD: Non-Communicable Diseases, Ref= reference category.

The self-reported diagnosis with hypertension, diabetes, cancer, chronic lung diseases, chronic heart diseases, stroke, arthritis, neurological problems, high cholesterol or other chronic conditions were included in assessment of NCD status. * - p-value <0.05, **- p-value <0.001. Note: The percentages reported are weighted percentages

Table 5 : Factors associated with CHE at households with female and male household heads (n =10963)

| **Variables** | **Female (n = 2129)** | **CHE** | **Male (n = 8834)** | **CHE** |
| --- | --- | --- | --- | --- |
|  | **%** | **AOR(95% CI)** | **%** | **AOR (95% CI)** |
| **Caste** |  |  |  |  |
| Scheduled caste | 49.2 | 1.26 (1.01 - 1.57)* | 44.1 | 1.03 (0.92 - 1.14) |
| Scheduled tribe | 34.1 | 0.78 (0.52 - 1.17) | 39.7 | 0.68 (0.57 - 0.82)** |
| Others | 45.3 | 1.41 (1.16 - 1.71)** | 36.4 | 0.83 (0.75 - 0.91)** |
| Other backward class (Ref) | 41.1 |  | 41.8 |  |
| **Income source** |  |  |  |  |
| Agricultural & non-agricultural business | 52.8 | 1.85 (1.52 - 2.24)** | 43.3 | 1.14 (1.05 - 1.24)* |
| Government subsidies & others | 50.0 | 1.76 (1.41 - 2.19)** | 51.7 | 1.85 (1.62 - 2.11)** |
| Individual salary (Ref) | 35.6 |  | 35.6 |  |
| **Wealth index** |  |  |  |  |
| Middle | 46.2 | 1.26 (1.01 - 1.56)* | 39.0 | 0.92 (0.82 - 1.02) |
| Poor | 44.9 | 1.24 (1.03 - 1.49)* | 40.1 | 1.1 (1.01 - 1.21)* |
| Rich (Ref) | 41.6 |  | 41.3 |  |
| **Residence** |  |  |  |  |
| Rural | 49.9 | 1.68 (1.40 - 2.03)** | 45.8 | 2.09 (1.90 - 2.29)** |
| Urban (Ref) | 35.4 |  | 28.4 |  |
| **Epidemiological transition level** |  |  |  |  |
| Low ETL | 49.7 | 1.25 (0.96 - 1.62) | 42.6 | 1.46 (1.28 - 1.66)** |
| Lower middle ETL | 19.4 | 0.34 (0.22 - 0.52)** | 24.7 | 0.74 (0.60 - 0.92)* |
| Higher middle ETL | 44.2 | 1.06 (0.84 - 1.35) | 42.5 | 1.53 (1.35 - 1.75)** |
| High ETL (Ref) | 41.7 |  | 34.0 |  |
| **Health insurance** |  |  |  |  |
| Government | 44.2 | 1.01 (0.63 - 1.63) | 37.7 | 1.29 (1.03 - 1.61)* |
| Not covered | 43.5 | 0.81 (0.52 - 1.26) | 41.9 | 1.54 (1.25 - 1.90)** |
| Private(Ref) | 44.4 |  | 28.6 |  |
| **NCD status** |  |  |  |  |
| NCD multimorbidity | 42.9 | 0.99 (0.84 - 1.17) | 43.1 | 1.38 (1.28 - 1.5)** |
| Single NCD (Ref) | 44.4 |  | 37.8 |  |
| **Type of care** |  |  |  |  |
| IP care | 47.3 | 1.98 (1.44 - 2.73)** | 38.4 | 1.39 (1.15 - 1.68)** |
| IP & OP care | 55.4 | 2.67 (2.17 - 3.27)** | 62.6 | 3.60 (3.28 - 3.94)** |
| OP care (Ref) | 39.1 |  | 33.0 |  |

**Dependent Variables:** Catastrophic Health Expenditure (CHE)= No (Ref), Yes;

ETL: Epidemiological Transition Level; **Low ETL**- Bihar, Jarkhand, Uttar Pradesh, Rajasthan, Meghalaya, Assam, Chhattisgarh, Madhya Pradesh, Odisha; **Lower Middle ETL**- Arunachal Pradesh, Mizoram, Nagaland, Uttarakahnd, Gujarat, Tripura, Manipur; **Higher Middle ETL**- Haryana, Delhi, Telangana, Andhra Pradesh, Jammu and Kashmir, Karnataka, West Bengal, Maharashtra, and Union territories other than Delhi; **High ETL**- Himachal Pradesh, Punjab, Tamil Nadu, Goa, and Kerala; HH=Household, IP= Inpatient, OP=Out Patient NCD: Non-Communicable Diseases, Ref= reference category.

The self-reported diagnosis with hypertension, diabetes, cancer, chronic lung diseases, chronic heart diseases, stroke, arthritis, neurological problems, high cholesterol or other chronic conditions were included in assessment of NCD status. * - p-value <0.05, **- p-value <0.001. Note: The percentages reported are weighted percentages

Table 6 : Factors associated with impoverishment at households with female and male household heads (n =10963)

| **Variables** | **Female (n = 2129)** | **Impoverishment** | **Male (n = 8834)** | **Impoverishment** |
| --- | --- | --- | --- | --- |
|  | **%** | **AOR(95% CI)** | **%** | **AOR (95% CI)** |
| **Caste** |  |  |  |  |
| Scheduled caste | 46.8 | 1.06 (0.81 - 1.39) | 36.1 | 1.19 (1.04 - 1.35)* |
| Scheduled tribe | 36.0 | 0.71 (0.43 - 1.16) | 35.5 | 1.21 (0.98 - 1.49) |
| Others | 26.7 | 0.85 (0.66 - 1.10) | 18.4 | 0.83 (0.73 - 0.94)* |
| Other backward class (Ref) | 33.8 |  | 27.3 |  |
| **Income source** |  |  |  |  |
| Agricultural & non-agricultural business | 43.1 | 2.65 (2.07 - 3.4)** | 29.1 | 1.38 (1.24 - 1.54)** |
| Government subsidies & others | 52.4 | 4.61 (3.48 - 6.11)** | 40.0 | 2.93 (2.49 - 3.45)** |
| Individual salary (Ref) | 21.5 |  | 21.6 |  |
| **Wealth index** |  |  |  |  |
| Middle | 35.1 | 24.32 (18.41 - 2.13)** | 19.1 | 22.87 (19.95 - 26.21)** |
| Poor | 63.2 | 4.44 (3.36 - 5.87)** | 55.0 | 3.34 (2.86 - 3.89)** |
| Rich (Ref) | 12.1 |  | 6.5 |  |
| **Residence** |  |  |  |  |
| Rural | 48.3 | 6.41 (4.95 - 8.3)** | 32.4 | 3.93 (3.46 - 4.46)** |
| Urban (Ref) | 15.6 |  | 13.6 |  |
| **Epidemiological transition level** |  |  |  |  |
| Low ETL | 43.2 | 0.86 (0.62 - 1.19) | 33.1 | 1.31 (1.11 - 1.56)* |
| Lower middle ETL | 19.4 | 0.58 (0.35 - 0.97)* | 12.8 | 0.73 (0.54 - 0.99)* |
| Higher middle ETL | 31.3 | 1.21 (0.89 - 1.64) | 24.4 | 1.27 (1.07 - 1.52)* |
| High ETL (Ref) | 33.2 |  | 19.2 |  |
| **Health insurance** |  |  |  |  |
| Government | 35.7 | 1.83 (0.93 - 3.63) | 26.0 | 1.42 (1.02 - 1.98)* |
| Not covered | 34.4 | 1.59 (0.82 - 3.06) | 27.4 | 1.41 (1.03 - 1.94)* |
| Private(Ref) | 18.9 |  | 13.6 |  |
| **NCD status** |  |  |  |  |
| NCD multimorbidity | 28.3 | 0.85 (0.69 - 1.05) | 22.7 | 0.98 (0.89 - 1.09) |
| Single NCD (Ref) | 39.9 |  | 30.3 |  |
| **Type of care** |  |  |  |  |
| IP care | 34.3 | 1.05 (0.69 - 1.59) | 25.2 | 1.14 (0.88 - 1.47) |
| IP & OP care | 26.7 | 1.19 (0.91 - 1.54) | 24.6 | 1.43 (1.27 - 1.61)** |
| OP care (Ref) | 36.8 |  | 27.3 |  |

**Dependent Variables:** Impoverishment=No (Ref), Yes.

ETL: Epidemiological Transition Level; **Low ETL**- Bihar, Jarkhand, Uttar Pradesh, Rajasthan, Meghalaya, Assam, Chhattisgarh, Madhya Pradesh, Odisha; **Lower Middle ETL**- Arunachal Pradesh, Mizoram, Nagaland, Uttarakahnd, Gujarat, Tripura, Manipur; **Higher Middle ETL**- Haryana, Delhi, Telangana, Andhra Pradesh, Jammu and Kashmir, Karnataka, West Bengal, Maharashtra, and Union territories other than Delhi; **High ETL**- Himachal Pradesh, Punjab, Tamil Nadu, Goa, and Kerala; HH=Household, IP= Inpatient, OP=Out Patient NCD: Non-Communicable Diseases, Ref= reference category.

The self-reported diagnosis with hypertension, diabetes, cancer, chronic lung diseases, chronic heart diseases, stroke, arthritis, neurological problems, high cholesterol or other chronic conditions were included in assessment of NCD status. * - p-value <0.05, **- p-value <0.001. Note: The percentages reported are weighted percentages

Table 7 : Factors associated with household economic conditions of female headed households (n = 2957)

| **Predictor variables** | **HH economic condition remained same** | | **HH economic condition Worsened** | |
| --- | --- | --- | --- | --- |
|  | **%** | **AOR (95% CI)** | **%** | **AOR(95% CI)** |
| **Caste** |  |  |  |  |
| Scheduled caste | 52.9% | 1.23 (0.97 - 1.56) | 24.9% | 1.4 (1.05 - 1.87)* |
| Scheduled tribe | 60.3% | 1.09 (0.74 - 1.59) | 11.6% | 0.47 (0.27 - 0.83)* |
| Others | 57.9% | 1.83 (1.48 - 2.26)** | 19.4% | 1.70 (1.30 - 2.23)** |
| Other backward class (Ref) | 51.3% |  | 20.2% |  |
| **Income source** |  |  |  |  |
| Agricultural & non-agricultural business | 56.7% | 1.51 (1.23 - 1.86)** | 24.2% | 2.36 (1.82 - 3.06)** |
| Government subsidies & benefits | 54.7% | 2.48 (1.89 - 3.25)** | 33.9% | 5.86 (4.29 - 8.01)** |
| Individual salary (Ref) | 52.0% |  | 12.1% |  |
| **Wealth index** |  |  |  |  |
| Middle | 63.4% | 2.32 (1.83 - 2.95)** | 18.6% | 1.95 (1.43 - 2.65)** |
| Poor | 56.4% | 2.42 (1.98 - 2.95)** | 25.0% | 3.37 (2.62 - 4.33)** |
| Rich (Ref) | 47.2% |  | 16.9% |  |
| **Residence** |  |  |  |  |
| Rural | 56.0% | 2.04 (1.68 - 2.46)** | 27.0% | 4.30 (3.34 - 5.53)** |
| Urban (Ref) | 51.2% |  | 11.2% |  |
| **Epidemiological transition level(ETL)** |  |  |  |  |
| Low ETL | 54.5% | 0.35 (0.26 - 0.47)** | 21.4% | 0.29 (0.20 - 0.42)** |
| Lower medium ETL | 55.9% | 0.42 (0.28 - 0.64)** | 18.4% | 0.5 (0.30 - 0.83)* |
| Higher medium ETL | 49.7% | 0.37 (0.28 - 0.48)** | 18.2% | 0.34 (0.25 - 0.46)** |
| High ETL (Ref) | 62.5% |  | 24.9% |  |
| **Health insurance** |  |  |  |  |
| Government | 63.2% | 0.94 (0.55 - 1.61) | 17.0% | 0.45 (0.23 - 0.88)* |
| Not covered | 51.0% | 0.65 (0.39 - 1.08) | 21.4% | 0.51 (0.27 - 0.97)* |
| Private (Ref) | 58.2% |  | 20.9% |  |
| **NCD status** |  |  |  |  |
| NCD multimorbidity | 52.5% | 1.02 (0.85 - 1.22) | 20.2% | 1.17 (0.93 - 1.46) |
| Single NCD (Ref) | 55.2% |  | 20.6% |  |
| **Type of care** |  |  |  |  |
| IP care | 44.3% | 0.64 (0.44 - 0.93)* | 11.4% | 0.45 (0.25 - 0.78)* |
| IP & OP care | 42.9% | 0.85 (0.65 - 1.12) | 19.7% | 1.28 (0.91 - 1.80) |
| OP care | 57.9% | 1.21 (0.97 - 1.51) | 20.5% | 1.15 (0.87 - 1.52) |
| No care (Ref) | 56.3% |  | 22.8% |  |

**Notes:** Dependent variable: Household Economic Condition in the previous two years: Improved (ref), remained same, worsened.

ETL: Epidemiological Transition Level; **Low ETL**- Bihar, Jarkhand, Uttar Pradesh, Rajasthan, Meghalaya, Assam, Chhattisgarh, Madhya Pradesh, Odisha; **Lower Middle ETL**- Arunachal Pradesh, Mizoram, Nagaland, Uttarakahnd, Gujarat, Tripura, Manipur; **Higher Middle ETL**- Haryana, Delhi, Telangana, Andhra Pradesh, Jammu and Kashmir, Karnataka, West Bengal, Maharashtra, and Union territories other than Delhi; **High ETL**- Himachal Pradesh, Punjab, Tamil Nadu, Goa, and Kerala; HH=Household, IP= Inpatient, OP=Out Patient NCD: Non-Communicable Diseases, Ref= reference category.

The self-reported diagnosis with hypertension, diabetes, cancer, chronic lung diseases, chronic heart diseases, stroke, arthritis, neurological problems, high cholesterol or other chronic conditions were included in assessment of NCD status. * - p-value <0.05, **- p-value <0.001.The percentages reported are weighted percentages.

Table 8 : Factors associated with household economic conditions of male headed households (n = 11110)

| **Predictor variables** | **HH economic condition remained same** | | **HH economic condition Worsened** | |
| --- | --- | --- | --- | --- |
|  | **%** | **AOR (95% CI)** | **%** | **AOR(95% CI)** |
| **Caste** |  |  |  |  |
| Scheduled caste | 54.6% | 1.33 (1.19 - 1.48)** | 20.4% | 1.75 (1.52 - 2.01)** |
| Scheduled tribe | 60.9% | 1.41 (1.20 - 1.67)** | 11.9% | 0.87 (0.68 - 1.12) |
| Others | 49.0% | 1.03 (0.94 - 1.12) | 14.3% | 1.10 (0.97 - 1.24) |
| Other backward class (Ref) | 52.1% |  | 14.4% |  |
| **Income source** |  |  |  |  |
| Agricultural & non-agricultural business | 54.1% | 1.14 (1.05 - 1.23)* | 15.6% | 1.16 (1.03 - 1.29)* |
| Government subsidies & benefits | 51.8% | 1.28 (1.12 - 1.45)** | 19.6% | 1.82 (1.54 - 2.15)** |
| Individual salary (Ref) | 50.6% |  | 14.2% |  |
| **Wealth index** |  |  |  |  |
| Middle | 50.7% | 1.08 (0.98 - 1.19) | 13.6% | 1.03 (0.89 - 1.19) |
| Poor | 55.8% | 1.57 (1.44 - 1.72)** | 17.2% | 1.85 (1.64 - 2.08)** |
| Rich (Ref) | 49.3% |  | 14.4% |  |
| **Residence** |  |  |  |  |
| Rural | 54.4% | 1.52 (1.39 - 1.65)** | 17.0% | 1.88 (1.66 - 2.12)** |
| Urban (Ref) | 47.5% |  | 11.9% |  |
| **Epidemiological transition level(ETL)** |  |  |  |  |
| Low ETL | 52.3% | 0.58 (0.51 - 0.66)** | 15.9% | 0.72 (0.61 - 0.86)** |
| Lower medium ETL | 50.6% | 0.67 (0.56 - 0.81)** | 16.9% | 1.02 (0.79 - 1.31) |
| Higher medium ETL | 49.6% | 0.57 (0.50 - 0.64)** | 14.8% | 0.70 (0.59 - 0.83)** |
| High ETL (Ref) | 60.1% |  | 14.7% |  |
| **Health insurance** |  |  |  |  |
| Government | 53.6% | 1.58 (1.29 - 1.93)** | 17.5% | 1.50 (1.13 - 1.99)* |
| Not covered | 52.2% | 1.45 (1.20 - 1.75)** | 14.8% | 1.14 (0.87 - 1.48) |
| Private (Ref) | 41.6% |  | 13.8% |  |
| **NCD status** |  |  |  |  |
| NCD multimorbidity | 50.3% | 0.97 (0.9 - 1.05) | 16.0% | 1.16 (1.04 - 1.29)* |
| Single NCD (Ref) | 53.8% |  | 14.7% |  |
| **Type of care** |  |  |  |  |
| IP care | 57.0% | 1.48 (1.19 - 1.83)** | 13.4% | 1.56 (1.13 - 2.14)* |
| IP & OP care | 49.2% | 1.23 (1.09 - 1.39)* | 20.0% | 2.33 (1.96 - 2.77)** |
| OP care | 52.7% | 1.22 (1.11 - 1.35)** | 15.0% | 1.57 (1.35 - 1.82)** |
| No care (Ref) | 52.5% |  | 11.7% |  |

**Notes:** Dependent variable: Household Economic Condition in the previous two years: Improved (ref), remained same, worsened.

ETL: Epidemiological Transition Level; **Low ETL**- Bihar, Jarkhand, Uttar Pradesh, Rajasthan, Meghalaya, Assam, Chhattisgarh, Madhya Pradesh, Odisha; **Lower Middle ETL**- Arunachal Pradesh, Mizoram, Nagaland, Uttarakahnd, Gujarat, Tripura, Manipur; **Higher Middle ETL**- Haryana, Delhi, Telangana, Andhra Pradesh, Jammu and Kashmir, Karnataka, West Bengal, Maharashtra, and Union territories other than Delhi; **High ETL**- Himachal Pradesh, Punjab, Tamil Nadu, Goa, and Kerala; HH=Household, IP= Inpatient, OP=Out Patient NCD: Non-Communicable Diseases, Ref= reference category.

The self-reported diagnosis with hypertension, diabetes, cancer, chronic lung diseases, chronic heart diseases, stroke, arthritis, neurological problems, high cholesterol or other chronic conditions were included in assessment of NCD status. * - p-value <0.05, **- p-value <0.001.The percentages reported are weighted percentages.

Table 9 : Factors associated with household economic conditions of households in rural (n = 8367)

| **Predictor variables/Rural** | **HH economic condition remained same** | | **HH economic condition Worsened** | |
| --- | --- | --- | --- | --- |
|  | **%** | **AOR (95% CI)** | **%** | **AOR(95% CI)** |
| **Gender of household head** |  |  |  |  |
| Female | 56.0% | 1.56 (1.37 - 1.77)** | 27.0% | 2.34 (2.01 - 2.72)** |
| Male (Ref) | 54.4% |  | 17.0% |  |
| **Caste** |  |  |  |  |
| Scheduled caste | 54.7% | 1.22 (1.09 - 1.38)** | 23.7% | 1.66 (1.44 - 1.92)** |
| Scheduled tribe | 60.0% | 1.13 (0.95 - 1.34) | 13.1% | 0.74 (0.58 - 0.94)* |
| Others | 51.5% | 0.88 (0.79 - 0.98)* | 17.8% | 0.93 (0.81 - 1.07) |
| Other backward class (Ref) | 55.6% |  | 17.7% |  |
| **Income source** |  |  |  |  |
| Agricultural & non-agricultural business | 55.7% | 1.21 (1.1 - 1.32)** | 17.7% | 1.3 (1.15 - 1.46)** |
| Government subsidies & benefits | 54.2% | 1.61 (1.38 - 1.87)** | 28.7% | 2.74 (2.29 - 3.27)** |
| Individual salary (Ref) | 53.7% |  | 16.6% |  |
| **Wealth index** |  |  |  |  |
| Middle | 57.7% | 1.34 (1.19 - 1.50)** | 16.6% | 1.11 (0.96 - 1.30) |
| Poor | 56.7% | 1.44 (1.3 - 1.60)** | 20.7% | 1.64 (1.44 - 1.87)** |
| Rich (Ref) | 51.1% |  | 18.0% |  |
| **Epidemiological transition level(ETL)** |  |  |  |  |
| Low ETL | 53.8% | 0.71 (0.62 - 0.82)** | 17.9% | 0.80 (0.67 - 0.97)* |
| Lower medium ETL | 49.4% | 0.59 (0.47 - 0.74)** | 18.5% | 0.83 (0.62 - 1.11) |
| Higher medium ETL | 54.4% | 0.82 (0.71 - 0.94)* | 19.8% | 0.98 (0.82 - 1.17) |
| High ETL (Ref) | 59.9% |  | 18.5% |  |
| **Health insurance** |  |  |  |  |
| Government | 56.4% | 1.11 (0.82 - 1.49) | 18.2% | 0.83 (0.57 - 1.2) |
| Not covered | 54.2% | 1.04 (0.78 - 1.39) | 18.9% | 0.80 (0.56 - 1.14) |
| Private (Ref) | 51.1% |  | 20.4% |  |
| **NCD status** |  |  |  |  |
| NCD multimorbidity | 52.8% | 0.96 (0.88 - 1.05) | 20.0% | 1.15 (1.03 - 1.29)* |
| Single NCD (Ref) | 56.1% |  | 17.8% |  |
| **Type of care** |  |  |  |  |
| IP care | 61.7% | 1.25 (0.96 - 1.63) | 16.9% | 1.15 (0.81 - 1.62) |
| IP & OP care | 48.4% | 0.80 (0.69 - 0.93)* | 22.5% | 1.38 (1.14 - 1.66)** |
| OP care | 54.9% | 0.90 (0.79 - 1.01) | 17.8% | 1.00 (0.85 - 1.18) |
| No care (Ref) | 59.4% |  | 18.1% |  |

**Notes:** Dependent variable: Household Economic Condition in the previous two years: Improved (ref), remained same, worsened.

ETL: Epidemiological Transition Level; **Low ETL**- Bihar, Jarkhand, Uttar Pradesh, Rajasthan, Meghalaya, Assam, Chhattisgarh, Madhya Pradesh, Odisha; **Lower Middle ETL**- Arunachal Pradesh, Mizoram, Nagaland, Uttarakahnd, Gujarat, Tripura, Manipur; **Higher Middle ETL**- Haryana, Delhi, Telangana, Andhra Pradesh, Jammu and Kashmir, Karnataka, West Bengal, Maharashtra, and Union territories other than Delhi; **High ETL**- Himachal Pradesh, Punjab, Tamil Nadu, Goa, and Kerala; HH=Household, IP= Inpatient, OP=Out Patient NCD: Non-Communicable Diseases, Ref= reference category.

The self-reported diagnosis with hypertension, diabetes, cancer, chronic lung diseases, chronic heart diseases, stroke, arthritis, neurological problems, high cholesterol or other chronic conditions were included in assessment of NCD status. * - p-value <0.05, **- p-value <0.001.The percentages reported are weighted percentages.

Table 10 : Factors associated with household economic conditions of households in urban (n = 5700)

| **Predictor variables** | **HH economic condition remained same** | | **HH economic condition Worsened** | |
| --- | --- | --- | --- | --- |
|  | **%** | **AOR (95% CI)** | **%** | **AOR(95% CI)** |
| **Gender of household head** |  |  |  |  |
| Female | 51.2% | 1.21 (1.06 - 1.38)* | 11.2% | 1.07 (0.87 - 1.31) |
| Male (Ref) | 47.5% |  | 11.9% |  |
| **Caste** |  |  |  |  |
| Scheduled caste | 52.5% | 1.58 (1.31 - 1.91)** | 12.3% | 1.48 (1.12 - 1.97)* |
| Scheduled tribe | 64.7% | 2.77 (1.97 - 3.90)** | 6.3% | 0.99 (0.51 - 1.92) |
| Others | 49.7% | 1.7 (1.50 - 1.92)** | 12.3% | 1.74 (1.43 - 2.11)** |
| Other backward class (Ref) | 45.0% |  | 11.5% |  |
| **Income source** |  |  |  |  |
| Agricultural & non-agricultural business | 50.0% | 1.18 (1.03 - 1.35)* | 14.0% | 1.48 (1.21 - 1.81)** |
| Government subsidies & benefits | 49.4% | 1.44 (1.19 - 1.74)** | 14.0% | 2.03 (1.55 - 2.67)** |
| Individual salary (Ref) | 47.6% |  | 10.5% |  |
| **Wealth index** |  |  |  |  |
| Middle | 54.5% | 1.09 (0.93 - 1.27) | 15.1% | 1.30 (1.02 - 1.67)* |
| Poor | 43.9% | 2.08 (1.82 - 2.37)** | 10.4% | 3.08 (2.51 - 3.78)** |
| Rich (Ref) | 44.4% |  | 9.1% |  |
| **Epidemiological transition level(ETL)** |  |  |  |  |
| Low ETL | 48.6% | 0.34 (0.28 - 0.42)** | 12.6% | 0.35 (0.27 - 0.46)** |
| Lower medium ETL | 54.4% | 0.54 (0.42 - 0.69)** | 15.7% | 0.65 (0.46 - 0.92)* |
| Higher medium ETL | 42.7% | 0.27 (0.23 - 0.32)** | 9.4% | 0.25 (0.19 - 0.32)** |
| High ETL (Ref) | 62.0% |  | 15.8% |  |
| **Health insurance** |  |  |  |  |
| Government | 53.1% | 2.04 (1.57 - 2.64)** | 15.3% | 2.05 (1.40 - 3.02)** |
| Not covered | 47.9% | 1.55 (1.24 - 1.95)** | 10.9% | 1.25 (0.88 - 1.78) |
| Private (Ref) | 39.6% |  | 11.3% |  |
| **NCD status** |  |  |  |  |
| NCD multimorbidity | 47.8% | 1.02 (0.9 - 1.14) | 12.2% | 1.13 (0.94 - 1.35) |
| Single NCD (Ref) | 49.1% |  | 11.2% |  |
| **Type of care** |  |  |  |  |
| IP care | 41.4% | 1.02 (0.77 - 1.35) | 7.1% | 0.92 (0.55 - 1.54) |
| IP & OP care | 47.2% | 1.73 (1.44 - 2.07)** | 14.8% | 3.05 (2.31 - 4.01)** |
| OP care | 50.9% | 1.66 (1.44 - 1.91)** | 12.3% | 2.03 (1.61 - 2.55)** |
| No care (Ref) | 44.8% |  | 9.1% |  |

**Notes:** Dependent variable: Household Economic Condition in the previous two years: Improved (ref), remained same, worsened.

ETL: Epidemiological Transition Level; **Low ETL**- Bihar, Jarkhand, Uttar Pradesh, Rajasthan, Meghalaya, Assam, Chhattisgarh, Madhya Pradesh, Odisha; **Lower Middle ETL**- Arunachal Pradesh, Mizoram, Nagaland, Uttarakahnd, Gujarat, Tripura, Manipur; **Higher Middle ETL**- Haryana, Delhi, Telangana, Andhra Pradesh, Jammu and Kashmir, Karnataka, West Bengal, Maharashtra, and Union territories other than Delhi; **High ETL**- Himachal Pradesh, Punjab, Tamil Nadu, Goa, and Kerala; HH=Household, IP= Inpatient, OP=Out Patient NCD: Non-Communicable Diseases, Ref= reference category.

The self-reported diagnosis with hypertension, diabetes, cancer, chronic lung diseases, chronic heart diseases, stroke, arthritis, neurological problems, high cholesterol or other chronic conditions were included in assessment of NCD status. * - p-value <0.05, **- p-value <0.001.The percentages reported are weighted percentages.
