# Supplementary material for: Financial impact of non-communicable diseases on households with older adults in india: a mixed methods study
Source: BMC Geriatr. 2026 Apr 18;26:772. doi: 10.1186/s12877-026-07493-9 (PMC13224535; doi:10.1186/s12877-026-07493-9)
Supplement: Supplementary file 3 — Supplementary Material 3. [file 12877_2026_7493_MOESM3_ESM.docx]

**Supplementary file 2 : Analysis of Catastrophic Health Expenditure at 30% and 25% Capacity to Pay cutoffs.**

We assessed the prevalence of catastrophic health expenditure (CHE) at three different capacity to pay (CTP) cut offs. These include the 40% CTP cut-off which is recommended for use in calculating CHE, and 30% and 25% CTP cut-offs. The lower CTP cutoffs yielded a higher prevalence of CHE (see table 1).

**Table 1:** Prevalence of Catastrophic Health Expenditure at various capacity to pay cutoffs.

| **CTP cutoff for CHE** | **Proportion of CHE** | **95% CI** |
| --- | --- | --- |
| 40% of CTP | 41.2% | 40.4% to 42.0% |
| 30% of CTP | 52.5% | 51.7% to 53.3% |
| 25% of CTP | 58.8% | 58.0% to 59.6% |

CTP= Catastrophic Health Expenditure, CHE= Catastrophic Health Expenditure, CI= Confidence Interval

We computed descriptive proportion of households incurring CHE at 30% and 25% cutoff across independent variables, and also computed adjusted odds ratios using binary logistic regression (see Table 2). The findings of the multivariate analysis for CHE-30 and CHE-25 also aligned with the associations identified with CHE at 40% cutoff (reported in Manuscript Table 3).

**Table 2:** Proportion of CHE across independent variables and factors associated with CHE at 30% and 25% of Capacity to pay cutoffs.

| **Independent Variables** | **CHE-30** | | **CHE-25** | |
| --- | --- | --- | --- | --- |
|  | **%** | **AOR(95% CI)** | **%** | **AOR(95% CI)** |
| **Gender of household head** |  |  |  |  |
| Female | 53.5 | 1.12 (1.03 - 1.23)* | 57.9 | 1.02 (0.93 - 1.12) |
| Male (Ref) | 52.0 |  | 58.7 |  |
| **Caste** |  |  |  |  |
| Scheduled caste | 55.0 | 1.00 (0.91 - 1.1) | 61.8 | 1.05 (0.95 - 1.16) |
| Scheduled tribe | 52.0 | 0.79 (0.68 - 0.93)* | 57.1 | 0.78 (0.67 - 0.91)* |
| Others | 50.2 | 1.02 (0.94 - 1.1) | 57.1 | 1.08 (1.00 - 1.18) |
| Other backward class (Ref) | 52.7 |  | 58.3 |  |
| **Income source** |  |  |  |  |
| Agricultural & non-agricultural business | 55.3 | 1.13 (1.05 - 1.22)* | 61.3 | 1.12 (1.04 - 1.21)* |
| Government subsidies & others | 61.7 | 1.67 (1.49 - 1.87)** | 67.3 | 1.64 (1.47 - 1.85)** |
| Individual salary (Ref) | 47.6 |  | 54.2 |  |
| **Wealth index** |  |  |  |  |
| Middle | 51.5 | 1.05 (0.96 - 1.15) | 58.2 | 1.10 (1.01 - 1.21)* |
| Poor | 53.7 | 1.30 (1.20 - 1.41)** | 60.6 | 1.38 (1.27 - 1.49)** |
| Rich (Ref) | 51.5 |  | 57.0 |  |
| **Residence** |  |  |  |  |
| Rural | 58.1 | 2.10 (1.94 - 2.28)** | 64.1 | 2.02 (1.87 - 2.19)** |
| Urban (Ref) | 40.5 |  | 47.3 |  |
| **Epidemiological transition level** |  |  |  |  |
| Low ETL | 54.6 | 1.20 (1.08 - 1.35)* | 61.2 | 1.24 (1.11 - 1.39)** |
| Lower middle ETL | 34.4 | 0.62 (0.52 - 0.73)** | 40.1 | 0.62 (0.52 - 0.74)** |
| Higher middle ETL | 54.0 | 1.24 (1.11 - 1.38)** | 60.1 | 1.25 (1.12 - 1.4)** |
| High ETL (Ref) | 48.0 |  | 53.9 |  |
| **Health insurance** |  |  |  |  |
| Government | 49.3 | 1.17 (0.97 - 1.41) | 56.9 | 1.16 (0.97 - 1.40) |
| Not covered | 53.7 | 1.37 (1.15 - 1.64)** | 59.5 | 1.25 (1.05 - 1.49)* |
| Private(Ref) | 41.4 |  | 49.3 |  |
| **NCD status** |  |  |  |  |
| NCD multimorbidity | 53.7 | 1.22 (1.13 - 1.3)** | 59.8 | 1.21 (1.13 - 1.30)** |
| Single NCD (Ref) | 50.9 |  | 57.3 |  |
| **Type of care** |  |  |  |  |
| IP care | 52.6 | 1.54 (1.32 - 1.81)** | 57.3 | 1.41 (1.21 - 1.66)** |
| IP & OP care | 72.9 | 3.59 (3.29 - 3.92)** | 77.1 | 3.40 (3.1 - 3.72)** |
| OP care (Ref) | 45.2 |  | 52.2 |  |

CTP= Catastrophic Health Expenditure, CHE= Catastrophic Health Expenditure, CI= Confidence Interval

Dependent Variables: Catastrophic Health Expenditure (CHE)= No (Ref), Yes.

HH=Household, IP= Inpatient, OP=Out Patient NCD: Non-Communicable Diseases, Ref= reference category.

The self-reported diagnosis with hypertension, diabetes, cancer, chronic lung diseases, chronic heart diseases, stroke, arthritis, neurological problems, high cholesterol or other chronic conditions were included in assessment of NCD status. * - p-value <0.05, **- p-value <0.001.The percentages reported are weighted percentages.
